# Genomic Features of a Food-Derived *Pseudomonas aeruginosa* Strain PAEM and Biofilm-Associated Gene Expression under a Marine Bacterial α-Galactosidase

**DOI:** 10.3390/ijms21207666

**Published:** 2020-10-16

**Authors:** Larissa Balabanova, Yuri Shkryl, Lubov Slepchenko, Daria Cheraneva, Anna Podvolotskaya, Irina Bakunina, Olga Nedashkovskaya, Oksana Son, Liudmila Tekutyeva

**Affiliations:** 1 Laboratory of Marine Biochemistry, G.B. Elyakov Pacific Institute of Bioorganic Chemistry, Far Eastern Branch, the Russian Academy of Sciences, 690022 Vladivostok, Russia; slepchenko.lubov@gmail.com (L.S.); buffy3000@mail.ru (D.C.); bakun@list.ru (I.B.); oned2004@mail.ru (O.N.); 2 Basic Department of Bioeconomy and Food Security, School of Economics and Management, Far Eastern Federal University, 690090 Vladivostok, Russia; apodvolot7777@mail.ru (A.P.); oksana_son@bk.ru (O.S.); tekuteva.la@dvfu.ru (L.T.); 3 Federal Scientific Center of the East Asia Terrestrial Biodiversity, Far Eastern Branch, the Russian Academy of Sciences, 690022 Vladivostok, Russia

**Keywords:** *Pseudomonas aeruginosa*, meat processing environment, comparative genomics, gene expression, biofilm, recombinant α-galactosidase, cold-shock protein

## Abstract

The biofilm-producing strains of *P. aeruginosa* colonize various surfaces, including food products and industry equipment that can cause serious human and animal health problems. The biofilms enable microorganisms to evolve the resistance to antibiotics and disinfectants. Analysis of the *P. aeruginosa* strain (serotype O6, sequence type 2502), isolated from an environment of meat processing (PAEM) during a ready-to-cook product storage (−20 °C), showed both the mosaic similarity and differences between free-living and clinical strains by their coding DNA sequences. Therefore, a cold shock protein (CspA) has been suggested for consideration of the evolution probability of the cold-adapted *P. aeruginosa* strains. In addition, the study of the action of cold-active enzymes from marine bacteria against the food-derived pathogen could contribute to the methods for controlling *P. aeruginosa* biofilms. The genes responsible for bacterial biofilm regulation are predominantly controlled by quorum sensing, and they directly or indirectly participate in the synthesis of extracellular polysaccharides, which are the main element of the intercellular matrix. The levels of expression for 14 biofilm-associated genes of the food-derived *P. aeruginosa* strain PAEM in the presence of different concentrations of the glycoside hydrolase of family 36, α-galactosidase α-PsGal, from the marine bacterium *Pseudoalteromonas* sp. KMM 701 were determined. The real-time PCR data clustered these genes into five groups according to the pattern of positive or negative regulation of their expression in response to the action of α-galactosidase. The results revealed a dose-dependent mechanism of the enzymatic effect on the PAEM biofilm synthesis and dispersal genes.

## 1. Introduction

With the growth of antibiotic resistance of pathogens, metabolites of marine bacteria with antimicrobial activity are of great interest for creating new approaches to combat them, because they may affect unknown metabolic pathways in the bacterial competitors [1,2]. In the marine bacteria *Cobetia amphilecti* KMM 296 and *Pseudoalteromonas* sp. KMM 701, candidates for the active metabolites may be hydrolytic enzymes [3,4]. When applying the highly active alkaline phosphatase CmAP from *C. amphilecti* KMM 296 or α-galactosidase α-PsGal from *Pseudoalteromonas* sp. KMM 701 on the mature biofilms of *P. aeruginosa*, *Salmonella enteritidis*, *Staphylococcus aureus*, and *Bacillus subtilis* isolated from the frozen ready-to-cook meat, a visible effect of their degradation was observed after a few hours of the enzymatic treatment [5]. The effect might be caused by the quorum sensing (QS)-mediated switching from the high to low (or contrarily) level of the biofilm-associated gene expression under the growth of bacterial cultures with the addition of the enzymes, or under the enzymatic treatment of their mature biofilms [3]. There are many studies focused on the strategy to inhibit or accelerate the bacterial cells’ physiological processes through disrupting or modulating their QS [2,6]. Currently, the work is underway to study the complete set of physiological changes that occur in bacteria and their communities during biofilm formation from the initial attachment to the microbial community and surfaces until returning to the planktonic lifestyle [7]. The multilevel hierarchical network of QS includes signaling and gene regulation events that are responsible for the production and reception of signaling molecules that result in the final outcome of communication—the regulation of target genes, whose products directly perform specific functions during cellular processes. When the concentration of signaling molecules reaches a threshold level, they activate cellular processes, which, as a rule, affect the expression of genes in the entire population. In *P. aeruginosa*, two acyl-homoserine lactone (AHL) QS regulatory systems, LasR-LasI and RhlR-RhlI, control directly or indirectly the expression of partially overlapping sets of genes involving primary signals (first messengers) [8,9]. The second messenger, intracellular signaling molecule of cyclic diguanylate monophosphate (c-di-GMP), is responsible for the synthesis of the extracellular matrix’s polysaccharides and proteins as the main metabolites for biofilm formation, as well as flagellum-dependent motility [10,11]. In *P. aeruginosa*, c-di-GMP stimulates the synthesis of two different extracellular polysaccharides Pel and Psl with multiple pathways of transcriptional control for the transition from the free-living bacteria to their sessile forms, i.e., formation of the biofilms with emphasizing the importance of Psl as a surface attachment determinant [12]. In turn, the Psl signaling involves two diguanylate cyclases (DGCs): SadC and SiaD to produce c-di-GMP [13,14]. The DGCs, SiaA and SiaD, were additionally found to promote cellular aggregation in response to detergent-associated stress, involving *psl* operon, during the *P. aeruginosa* PAO1 growth with toxic Na^+^-dodecyl sulfate (SDS) [15]. SadC and RoeA modulate the transition from reversible to irreversible attachment via the regulation of flagellar reversal rates, swarming motility, and Pel polysaccharide production, respectively [13]. Consequently, bacteria may contain sets of site-specific DGSs and phosphodiesterases (PDEs), which are involved in the metabolism of c-di-GMP and various cell-signaling events [16,17,18]. The catalytic signature motifs GGDEF, HD-GYP (for DGCs), and EAL, DDFGTG (for PDEs), as well as sensory and signal transduction domains (PAS, PAC, HAMP, GAF, PBPb, REC and CHASE) mediating signal transduction are typical for the proteins from the c-di-GMP system. However, the identification of proteins that function as receptors/effectors for the c-di-GMP system has proven to be more complex. It is known that c-di-GMP can bind to various classes of proteins that differ in structure and amino acid sequences [16,17,19].

Despite the fact that the c-di-GMP signaling molecule regulates the transition between the mobile and sessile bacterial forms, many environmental signals and their mechanisms of action, triggering the formation of biofilms, remain unclear. It has been shown that individual amino acids contribute to the formation of biofilms through c-di-GMP signaling in artificial conditions [20]. The small molecule modulators, such as nitric oxide (NO), stimulating PDE activity, and bacterial matrix-degrading enzymes can induce the dispersal of *P. aeruginosa* biofilms [7,21,22]. However, either the c-di-GMP signaling pathway is complex for the development of molecular modulators or some strategies of extracellular matrix degradation contribute to disseminating bacteria with enhanced colonization properties [21].

The genetic regulation of biofilm growth, remodeling, and dispersal depending on the bacterial response to local changes in the environmental conditions underlie these processes. The species- and strain-specific genes in a biofilm or individual clone may be responsible for the QS and biofilm formation depending on the adaptation, survival, and dispersion strategy inherent of the individual *P. aeruginosa* lifestyle [8,9]. However, the novel environmental inputs and mechanism of the gene expression under their action in *P. aeruginosa* is still under elucidation. To date, the whole genomes of more than 4500 *P. aeruginosa* clones are sequenced, mainly of clinical or host-associated samples, which makes it possible to analyze the key genes of various physiological processes in the cells, including in the novel environmental *P. aeruginosa* isolates [23].

## 2. Results and Discussion

### 2.1. Whole Genome Sequencing and Annotations

**Figure 1:**
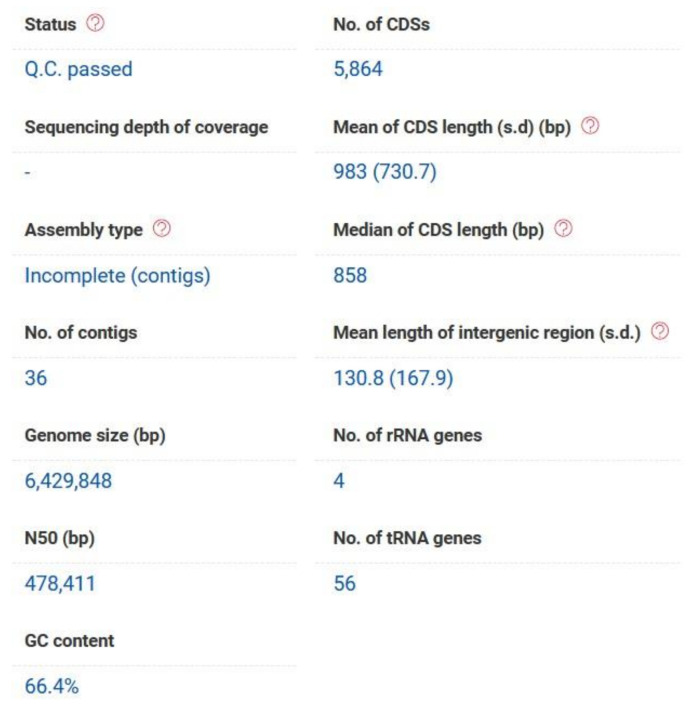
The whole-genome assembly (WGA) features of the *P. aeruginosa* strain PAEM calculated with the use of EzBioCloud genome annotation pipeline (www.ezbiocloud.net/). The server processed the contigs with the length of no less than 500 bp.

The whole genome shotgun sequencing (WGS) project for the *P. aeruginosa* strain PAEM (6.4 Mb) isolated from a frozen ready-to-cook meat (the environment of meat (EM) processing) has been deposited at DDBJ/ENA/GenBank under the accession JACBNI010000000 (BioProject: PRJNA644471). The genome sequence has been annotated by the NCBI Prokaryotic Genome Annotation Pipeline (PGAP; Annotation Method: Best-placed reference protein set; GeneMarkS-2). A total of 5986 genes have been identified, including 5884 protein-coding sequences (CDSs) and 62 genes for RNA (3 rRNA, 55 tRNA, 4 ncRNA). The pseudogenes (40) contain frameshifted (27), incomplete (16), internal stop (6), and multiple problems (7) genes. The genome contains three Clustered Regularly Interspaced Short Palindromic Repeat (CRISPR) arrays (JACBNI010000000). In respect that the large portion of *P. aeruginosa* CDSs are not functionally verified and, therefore, annotated as hypothetical proteins, they have been additionally analyzed through the RAST (Rapid Annotation Sequence Tool) and EzBioCloud servers [24,25]. Orthologous Average Nucleotide Identity (OrthoANIu) is the standard algorithm used to build the EzBioCloud database [26]. The *P. aeruginosa* PAEM genome (6.4 Mb) has a G+C content of 66.4%, and the assemblies contain 6185 (207 contigs with protein-encoding genes, PEG), and 5864 (36 contigs with PEG of more than 500 bp) potential CDSs with the bacterial core genes coverage 98.9% according to the RAST and EzBiocloud annotations, respectively (Figure 1, Table S1).

There are 33% CDSs (1994 genes including 109 hypothetical) with the functional features and 67% CDSs (4191 genes including 1531 hypothetical) non-belonging to the metabolic pathways identified by the RAST Subsystems [24]. The prevalent number of genes belongs to such functional categories as amino acids and derivatives (550), carbohydrates (290), membrane transport (261), and protein metabolism (231). The category “Secondary metabolism” includes the genes related to plant hormones (auxin biosynthesis) and bacterial cytostatics (paerucumarin biosynthesis) (data not shown). The results from the *classifier Levofloxacin* has identified three genome regions responsible for the predicted levofloxacin sensitivity in *P. aeruginosa* PAEM [24]. The predicted regions 1 and 2 were found to be identical with the genome sequences of the reference strain *P. aeruginosa* PAO1 by the DNA Motif Search tool in the current Pseudomonas Genome Database (DB) [23]. The genome examination for the presence of resistance genes by RAST found 30 CDSs for colicin E2, polymyxin, vancomycin, fosfomycin, and multidrug resistance proteins, including responsibility for heavy metals, cobalt–zinc–cadmium, chromium, copper, tellurium, and organic hydroperoxide, which are used in some industrial processes. The protectants against oxidative damage and other stress factors (85 searching results) are encoded by a multitude of stress response regulators, such as sodium hydrogen antiporters, outer membrane stress sensor proteases (proteolysis and periplasmic stress response), SOS—and NO—response regulators, uptake regulators of free iron and heme, paraquat-inducible proteins, alkyl hydroperoxide reductases, starvation, osmotic, temperature response regulators, and DNA repair proteins (44 results) [24].

According to the EzBiocloud annotation [25], there are 1706 genes encoding hypothetical proteins and about 158 results related to CDSs with multidrug resistance, stress response, or antibiotic biosynthesis functions (by EzBioCloud similarity) (Tables S1–S3). These include the resistance to neomycin, paromomycin, ribostamycin, butirosin, gentamicin B, trimethoprim, methotrexate, azomycin, acriflavin, antimicrobial lipopeptide surfactants, puromycin, tetraphenylarsonium chloride, mycotoxin fusaric acid, sulfonamide bicyclomycin, chloramphenicol, carbenicillin, erythromycin, novobiocin, streptomycin, tetracycline, macrolides–lincosamides–streptogramin B (MLS), teicoplanin, antimicrobial peptides (colistin, bacitracin, polymyxin), beta-lactams (methicillin and carbapenem resistance), the herbicide dinoseb, metronidazole, carbonyl cyanide m-chlorophenyl-hydrazone (CCCP), carbonyl cyanide 4-trifluoromethoxyphenylhydrazone (FCCP), 2,4- dinitrophenol (DNP), nalidixic acid, fosfomycin, benzalkonium chloride, and fluoroquinolones (Tables S1 and S2). Apart from the predicted resistance to most antibiotics, the strain PAEM possesses the genes responsible for resistance against broad-spectrum antiseptics and damaging environmental factors, including the resistance to ethidium bromide and quaternary ammonium compounds, organic hydroperoxides, arsenic, polyamines, acids (the acidic stomach environment, high levels of acetic acid, dichloroacetic acid), tributyltin chloride, toluene, phenol, alcohol, cumene hydroperoxide, and dichloroacetic acid (glutathione-dependent dehalogenation) (Tables S1 and S2). About 44 multidrug-resistance and 87 CDSs for stress-response proteins were found. Numerous CDSs related to chaperones (62 CDSs); cold shock CapA, CspA, CapB (6 CDSs); heat shock (19 CDSs) and phage shock proteins, holin-like proteins, and other osmoprotectants (40 CDSs); miniconductance mechanosensitive channels (against hypoosmotic shock); sodium, potassium, lithium, rubidium, and organophosphate Pi antiporters, chloride/bicarbonate exchanger (25 CDSs); photoreceptors and light response regulators (25 CDSs), DNA damage protectors (72 CDSs) and antiviral defense factors, such as CRISPR-associated proteins (7 CDSs) (Tables S1 and S2). The antibiotic biosynthesis genes of PAEM are similar by the genes from the reference strain PAO1 with 99.2–99.7% of identity, excluding the activator of bacteriocin biosynthesis PrtN (97.7%) (Table S3).

Transcriptional regulators and their regulatory proteins related to quorum sensing (17 CDSs), biofilm formation (16 CDSs), and polysaccharide metabolism/catabolism (70 CDSs) have also been identified as being involved in the resistance to damaging environment factors, as well as in the virulence and pathogenicity of *P. aeruginosa* PAEM, according to the EzbioCloud searching results (Table S4). However, many of the diguanilate cyclases (16 CDSs), histidine kinases, and other two-component response regulators (102 CDSs) are also known to be involved in the processes of cell adhesion, formation, maturation, and degradation of the *P. aeruginosa* biofilm (Table S5).

### 2.2. Comparative Pathogenomics, Serotyping, and Multilocus Sequence Typing

Searching for virulence and pathogenicity factors by the EzBiocloud similarity resulted in 80 and six CDSs, respectively (Table S1). However, the comparative pathogenomics with the use of database VFPB (Virulence Factors of Pathogenic Bacteria, http://www.mgc.ac.cn/VFs/) revealed that the strain PAEM from the environment of meat processing has the similar sets of genes; most of which are of a high level of mosaic identity (average 99.2–100%) with the corresponding sequences of the reference strains *P. aeruginosa* PAO1 and LESB58 (Table S6). They include all the known *P. aeruginosa* virulence factors, namely: 46 flagella genes (*flg*, *fli*, *fla*, *flh*, *mot*) related to the adherence; 33 genes (*pil*, *fim*, *chp*) of the type IV pili and twitching motility proteins biosynthesis; 11 genes of the lipopolysaccharide (LPS) O-antigen biosynthesis; 16 genes (*phz*) related to antimicrobial activity (phenazines biosynthesis pathway); 24 genes (*alg*, *muc*) related to antyphagocytosis (alginate biosynthesis and regulation); 3 genes (*rhl*) related to biosurfactants (rhamnolipid biosynthesis); 4 genes (*plc*, *pld*) of hemolytic and non-hemolytic phospholipases C and D; 26 genes (*pch*, *fpt*, *pvd*) for iron uptake (pyochelin, pyoverdin, and their receptors); 4 genes (*aprA*, *lasA* and *B*, *prpL*) of proteases (alkaline phosphatase, elastase, and protease IV); 7 genes (*hdtS*, *lasR* and *lasl*, *ahlR* and *ahll*, *rhll*) related to quorum sensing; the sensor kinase’s, GacS, and its response regulator’s, GacA, (GacS/GacA) two-component system genes *gacS* and *gacA*; 20 genes related to Hcp secretion island-1 encoding type VI secretion system (H-T6SS); 35 genes of the *P. aeruginosa* type III secretion system (TTSS) and 3 genes related to the *P. aeruginosa* TTSS translocated effectors (*exo S*, *T* and *Y*); exotoxin-A (*toxA*); and three genes (*hcn*A, B and C) for hydrogen cyanide production (Table S6). The environmental strain PAEM mostly distinguishes from the both comparative strains PAO1 and LESB58 by the genes of QS-dependent protease IV *prpL* (98.6 and 98.2%, respectively), elastase *lasA* (98.8 and 99.4%), QS-related genes for acylhomoserine lactone synthase *htdS* (99.2 and 99.1%), N-(butanoyl)-L-homoserine lactone QS system *rhll* (98.6 and 99.5%) and *rhlR* (99.6 and 99.7%), two-component system *gacS* (99.7%), sulfate transporter *pcrV* from TTSS (99.7 and 99.4%, respectively), (ABC)-importer for iron acquisition *pchI* (98.3 and 98.4%), pyoverdine-related *pvdD* (70.0 and 99.9%), *pvdJ* (73.8 and 99.8%) and *pvdY* (99.1 and 99.3%), rhamnosyltransferase (aclacinomycin-T 2-deoxy-L-fucose transferase, by EzBiocloud) *rhlB* (99.3 and 99.5%), exotoxin *toxA* (99.5 and 99.0%), exoenzymes *exoS* (99.3 and 100%), *exoT* (98.8 and 99.0%) and *exoY* (99.4 and 99.6%), and alginate regulation *mucD* (99.9 and 99.2%) and *algW* (99.8 and 99.3%) (Table S6).

The differences in such category of virulence factors as LPS O-antigens are also observed among the strains *P. aeruginosa* PAO1, LESB58, and PAEM (Table S6). No hits were found in the sequences of the strains LESB58 (serotype O6) and PAEM against the Wzx/Wzy-dependent pathway genes (*wbpA*, *wbpB*, *wbpE*, *wbpD*, and *wbpI*), which are essential for B-band O-antigen biosynthesis in *P. aeruginosa* serotype O5 (PAO1) and O16 [27,28]. The O-antigen biosynthesis gene cluster of the PAEM strain was successfully aligned to the *P. aeruginosa* serotype O6 O-antigen biosynthesis cluster (GenBank ID: AF498417.1) with the identity 99.9 at 99.97% of coverage [29].

The multilocus sequence typing (MLST) by the “mlst” software with the use of the *P. aeruginosa* PubMLST database (https://pubmlst.org/) attributed the PAEM genes (*acsA*, *aroE*, *guaA*, *mutL*, *nuoD*, *ppsA*, and *trpE*) to the sequence type (ST) 2502, whose clonal complex is not defined [30]. Two records belonging to the unpublished isolates from a bottled natural mineral water in Brazil (2018) and from a dog with otitis in France (2010) were found in the *P. aeruginosa* PubMLST database. The phylogenetically closest STs were from two isolates of STs 131 and 957 (UK) and one isolate of ST 663 from France [30].

The environmental *P. aeruginosa* PAEM possesses the most abundant CRISPR/Cas system of the type I-F, an antiviral adaptive immune system conferring resistance to foreign genetic elements from plasmids and phages [31]. This type was shown to correlate strongly with the susceptibility of the strain to multiple clinically relevant antibiotics for the prevalent STs 111 and 235 [31].

### 2.3. Comparative Genomics and Singletons Analysis

**Figure 2:**
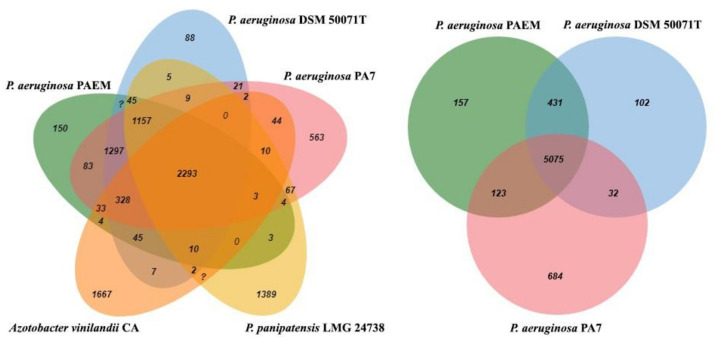
Venn diagram displays overlaps between *P. aeruginosa* PAEM, *P. aeruginosa* DSM 50071^T^, *P. aeruginosa* PA7 (right), *P. panipatensis* LMG 24738^T^, and *A. vinilandii* CA (left). The diagram was generated by EzBioCloud CG [25].

**Figure 3:**
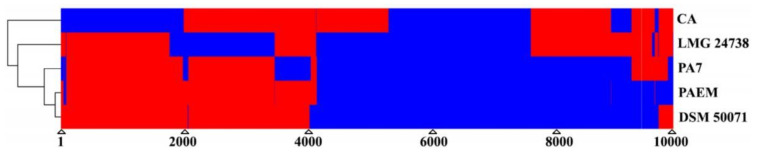
Coding genes content (presence/absence) data on the *P. aeruginosa* **PAEM**, *P. aeruginosa* **DSM 50071^T^**, *P. aeruginosa* **PA7**, *P. panipatensis* **LMG 24738**^T^, and *A. vinilandii* **CA** genomes clustered by the UPGMA algorithm (Unweighted Pair Group Method with Arithmetic mean). All CDSs are clustered into pan-genome orthologous groups (POGs). POG contains at least one protein-coding sequences (CDS) (called singleton POGs) and highly conserved POGs are found in all genomes, which comprises the core genome. The heat map is generated by EzBioCloud CG [25].

The strains *P. aeruginosa* DSM 50071^T^, *P. aeruginosa* PA7, *Pseudomonas panipatensis* LMG 24738, and *Azotobacter vinilandii* CA were selected and analyzed by the EzBiocloud Comparative Genomics (CG) (Table S7, Figure 2 and Figure 3). The common 2293 genes and 5075 of the *P. aeruginosa* core genes were found in the sampling (Figure 2 and Figure 3). Although *P. aeruginosa* PAEM has the expected core and pangenome, it revealed 150 and 157 singletons aligned against the orthologues and *P. aeruginosa* strains, respectively (Figure 2 and Figure 3). In addition, the PAEM singletones have either low identity or no homologues when aligned against the reference *P. aeruginosa* PAO1 or LESB58 sequences by the Basic Local Alignment Search Tool (BLAST) in the Pseudomonas Genome DB (http://www.pseudomonas.com/). The PAEM singletons found by the EzBioCloud CG tools (Table S7) without any similarity with the reference strains *P. aeruginosa* DSM 50071^T^, PA7, PAO1 and LESB58 have been additionally analyzed by the BLAST programs in the National Center for Biotechnology Information (NCBI) and Pseudomonas Genome DB (https://www.ncbi.nlm.nih.gov/; http://www.pseudomonas.com/).

There were no best reciprocal blast hits found for the CDSs for RNA-directed DNA polymerase PAEM_00124 (Tables S1 and S7) with the exception of an urine-derived isolate (95.8%) of carbapenem-resistant *P. aeruginosa* BH9 from Brazil (GenBank: CP029713.1) and *Sinimarinibacterium* sp. NLF-5-8 (93%) isolated from the wastewater treatment plants for livestock in South Korea (GenBank: CP048030.1). However, the reference non-redundant protein sequence (RefSeq: WP_023102177.1) containing isolates of *P. aeruginosa* from the human bloodstream, urogenital and eye infections, as well as of the foodborne pathogen *Salmonella enterica* isolated from ground chicken (USA) (GenBank: EAU0154325.1), the clinical isolates of *Alcaligenes faecalis* from wound, and several strains (*Spongiibacter tropicus*, *Rheinheimera* sp., *Haliea* sp.), with metagenome-assembled genomes, from the Pacific ocean were identical with PAEM_00124 by 100, 97, 95, and 87%, respectively.

The DNA polymerase IV (EC 2.7), similar to PAEM_00120 (Tables S1 and S7), is widely distributed among the intestinal bacteria *P. putida* and *P. fluorescence* (NCBI Multispecies Reference Sequence (RefSeq): WP_003160626.1) [32]. However, this record of five hypotetical proteins identical to PAEM_00120 include *P. aeruginosa* BL12 from the eye infection (GenBank: ERY33665.1) and the multidrug-resistant (MDR) Japanese strain of *P. aeruginosa* NCGM1179 (GenBank: GAA18645.1) from the respiratory tract. The strain NCGM1179 was highly resistant to carbapenems, aminoglycosides, and fluoroquinolones [33].

The singleton PAEM_00125 (Tables S1 and S7) with the predicted function of the XRE family transcriptional regulator or master regulator for biofilm formation (by EzBioCloud annotation) is identical to NCBI RefSeq WP_023102178.1 that corresponds to five records for *P. aeruginosa*, including the same clinical strains described above and one crude oil-degrading isolate from the Gulf of Mexico (GeneBank: RFK48079.1).

The adenine-specific DNA-methyltransferase PAEM_00130 (Tables S1 and S7), belonging to the type I restriction system, did not find the identical CDSs, but its protein sequence has the identity of 96–100% with the proteins from the same *P. aeruginosa* isolates (RefSeq WP_079265932.1).

The probable tyrosine recombinase PAEM_00144 in *P. aeruginosa* (Tables S1 and S7) is of 100% protein identity with RefSeq WP_003160612.1, which includes the Japanese MDR clinical isolate NCGM1179. The PAEM singletons encoding for the itegrases/recombinases PAEM_00143, PAEM_00145, PAEM_04638 do not have any similarity, as well as orthologues among Pseudomonadaceae, according to the NCBI and EzBioCloud (EggNog/COG) databases (Tables S1 and S7). In addition, the nucleotide and protein sequences of PAEM_00143, PAEM_00144 are well aligned against the sequences of *Comamonas testosteroni* P19 isolated from the biphenyl as the sole carbon source from the waste water of South Korea (GenBank: LN879547).

The singleton PAEM_00412 (Tables S1 and S7), encoding for the predicted tRNA(fMet)-specific endonuclease VapC2, a toxic component of a toxin–antitoxin (TA) system, was aligned against the multispecies RefSeq WP_003105740.1, including the putative nucleic acid binding proteins of *P. aeruginosa* (GenBank: YP_006960443.1; AGO43171.1), endonuclease VapC for *Salmonella enterica* (GenBank: ECA4546738.1), or hypothetical proteins for such strains as *P. aeruginosa* PABE173_5365 isolated from a glass-crusher air plate (GenBank: EKA41331.1) and BL12 from the eye infection (GenBank: ERY24639.1). The antitoxin component VapB2 (PAEM_00413) of the (TA)-module has the lower number of identical sequences (Tables S1 and S7). Among 24 genes from the 4662 genome assemblies of *P. aeruginosa* aligned against PAEM_00413 (VapB2) in the specific Pseudomonas Genome DB, only 12 genes from clinical isolates were without mismatches.

The singleton PAEM_00443 and PAEM_00444 (Tables S1 and S7), encoding for the reparative/replicative DNA helicase of subfamily UvrD/REP and hypothetical DNA repair protein RecF, respectively, found the nucleotide matches (100%) with only the *P. fluorescence* strain NCTC10783 isolated from the respiratory tract (GenBank LR134300.1), but with 99.8% of the protein identity with the multispecies AAA family ATPase [Pseudomonas] (NCBI RefSeq: WP_003125302.1) that include again the clinical isolates *P. aeruginosa* B12, NCMG1179, and 3575.

From 341,478 to 347,944 nucleotides (Tables S1 and S7), there are nine PAEM-specific CDSs for hypothetical proteins related to the family RebB (Pfam: Family 11747) killing trait of the endosymbiont bacteria, methyl-accepting chemotaxis protein, RNA polymerase sigma factors, and cAMP-dependent regulator, which have orthologues in all available species from EggNOG (evolutionary genealogy of genes: Non-supervised Orthologous Groups).

The extended clusters of PAEM singletons from c(426058..428253) to c(445219..445413) in the contig 15 (Tables S1 and S7) include 13 hypothetical proteins predicted as adenine-specific DNA-methyltransferase (type II restriction system, defense mechanism), multispecies transposases, and TonB-dependent siderophore-related receptor. The hypothetical proteins PAEM_03139 and PAEM_03140 also relate to the probable TonB-dependent receptors, which are similar to the PAO1 and LESB58 hypothetical proteins (99.0 and 99.6%). From c(420623..421225) to c(423573..424352) in the contig 21 (Tables S1 and S7), there are four hypothetical proteins with no predicted function and orthologues, but with the identity to hypothetical proteins from the *P. aeruginosa* LESlike isolates (data not shown).

The protein RelA (PAEM_03870), containing the regulatory domain of the bifunctional enzyme SpoT (Pfam PF0467), predicted as GTP diphosphokinase (absent in PAO1 and LESB58) has 100% identity with six reference sequences of the *P. aeruginosa* (p)ppGpp synthetase or GTP pyrophosphokinase (RefSeq: WP_079393115.1). In eubacteria, ppGpp (guanosine 3′-diphosphate 5-′diphosphate) is a mediator of the stringent response that coordinates a variety of cellular activities in response to changes in nutritional abundance [34].

The singleton PAEM_03874 (Pyocin-S2) (Tables S1 and S7), belonging to the colicin/pyocin nuclease family, has 98% identity with the homing endonuclease from the structural family HNH (Pfam CL0263) of *Salmonella enterica* (GenBank: ECA4547102.1), 100% with *P. aeruginosa* RefSeq (WP_144126122.1, WP_148711685.1, WP_121379800.1, ERU77283.1), and 99.4% with the PAO1 strain (absent in LESB58). In addition, the HNH nuclease PAEM_04208 (Tables S1 and S7) is identical by 100% to the PAO1 hypothetical protein PA0820 (absent in LES58). Pyocins belong to a larger group of bacteriocins, which are used by bacteria to compete for resources by killing competitors, usually of the same bacterial species [34].

The singletons PAEM_03937 and PAEM_03938 (Tables S1 and S7) were predicted to have the putative salinity stress responsive phosphoserine phosphatase (M:COG1213) or sugar nucleotidyltransferase (POG001052) functions, which are of 99.3–99.5 and 99.0–99.2% identity to the hypothetical proteins in PAO1 and LESB58, respectively (Tables S1 and S7). However, the hypothetical protein PAEM_04645 (Tables S1 and S7), which is related to a metallo-beta-lactamase domain protein according to EggNOG function, was found to have no matches within the comparative genomes suggested by EzBioCloud, as well as with PAO1 and LESB58. The CDS homologues of 100% identity were found in the hospital strains *P. aeruginosa* DVT401 (GenBank: CP050335) and DVT 425 (GenBank: CP050325), and *P. fluorescens* NCTC10783 (GenBank: LR134300). It has 100% amino acid identity with the multispecies metallo-beta-lactamase (MBL) fold hydrolase [Pseudomonas] (NCBI RefSeq: WP_003126469.1), including the environmental and host-associated *P. aeruginosa* strains, such as ATCC 25324 from air from the glass-crusher (GenBank: EKA34652.1), BL12 from the eye infection (GenBank: ERY39277.1), and the human isolate 3575 (GenBank: EZO25326.1). Only two CDSs for the MBL fold metallo-hydrolase from the human isolate *P. aeruginosa* N15-01092 (GenBank: CP012901.1) and sheep mastitis PSE305 (GenBank: HG974234) were found in the Pseudomonas Genome DB with 92.2% of identity (http://www.pseudomonas.com/).

PAEM_04659 encoding for the 2-hydroxymuconate (4-oxalocrotonate) tautomerase family protein, related to the metabolism of aromatic compounds, particularly aromatic amines, has no matches within the DSM 500071^T^ (Table S7), PAO1, and LESB58 genomes, but 100% identity with the multispecies protein [Pseudomonas] (RefSeq: WP_003159697.1), including 13 sequences from the MDR *P. aeruginosa* strains BL12, BL24, BWHPSA003, BWHPSA017, CF18, X24509, Z61, 3575, BWH050, BWH055, NCMG1179, *Pseudomonas knackmussii*, and *Acinetobacter baumannii*, which degrade chloroaromatic compounds [35,36].

The gene cluster containing the putative stress-responsible OsmC-like porin, dihydrolipoyllysine-residue acetyltransferase PAEM_04661 (Tables S1 and S7); disulfide-isomerase PAEM_04662 (putative polyketide synthase from 25 strains of *P. aeruginosa*, particularly involved in the aromatic antibiotic production, such as isochromanequinones frenolicin and nanaomycins); the tetR family transcriptional regulator-like protein PAEM_04665, negative regulator of growth toxin ParE (induces the SOS response, inhibits cell division, and promotes biofilm formation) aligned against nine hypothetical proteins from the listed above strains (MULTISPECIES: [Pseudomonas] Ref Seq: WP_003125306.1; WP_015060171.1; WP_003125102.1).

From (197,990...198,724) to (203,991...204,881) bases (Tables S1 and S7), there is a locus of seven PAEM singletons encoding for tRNA-dependent cyclodipeptide synthase proX—PAEM_05797 (MULTISPECIES: tRNA-dependent cyclodipeptide synthase [Pseudomonas] Ref Seq: WP_003158562.1) and five paralogues of hypothetical proteins with the EggNog function of 2OG-Fe(II) oxygenase superfamily, namely the multiply duplicated isopenicillin N synthase family oxygenase—PAEM_05798, PAEM_05799, PAEM_05801, PAEM_05802 (Ref Seq: WP_023132409.1), and gibberellin 3-beta-dioxygenase PAEM_05803 (Tables S1 and S7). The identical proteins exist in many environmental strains, including the sewage *P. aeruginosa* ENV-208, as well as the rhizosphere *P. aeruginosa* M18 [37]. The available orthologues were found in *Photorhabdus luminescens* subsp. *laumondii* TTO1, the marine gamma proteobacterium HTCC2080, and *Photorhabdus asymbiotica* subsp. *asymbiotica* ATCC 43949 (data not shown). The evolutionary significance of paralogues or gene duplication is to increase the likelihood of their expression and thus to strengthen the reliability of signs in the coding in which they participate. On the other hand, repeatedly duplicated genes probably serve as a “storage” of mutations that are harmful for the current environmental conditions, but giving the evolutionary advantage of any other conditions. Thus, when environmental conditions change quickly, organisms can “find” the most beneficial mutations from an accumulated set under these conditions and therefore quickly adapt to them [37].

One more PAEM singleton locus contains the Type-I restriction system S of methylase family *hsdM* (PAEM_05832), type I site-specific deoxyribonucleases *hsdS* (PAEM_05832) and *hsdR* (PAEM_05834), RNA-directed DNA polymerase (PAEM_05836) and hypothetical proteins PAEM_05833, PAEM_05835, and PAEM_05837, which putatively also related to the restriction-modification_system (Tables S1 and S7). Only *hsdS* found six matches in NCBI with 100% identity with the restriction endonuclease subunit S from the carbapenem- and colistin-degrading *P. aeruginosa* (Ref Seq: WP_079386772.1). In Pseudomonas Genome DB, 30 hits were found against PAEM_05832 (*hsdS*), including the veterinary strain VET-48 from the dog’s ear secretion (GenBank: RAED01000237).

### 2.4. Genomic Islands Analysis

Genomic islands (GIs) are commonly defined as clusters of genes of probable horizontal origin in bacterial or archaeal genomes. They often provide adaptive traits that enhance the fitness of a microorganism within a niche, encoding novel genes, environmentally relevant adaptations such as metal resistance, and entire metabolic pathways, such as the degradation of monoaromatic hydrocarbons. One of the important goals of GI predictions is pathogen outbreak analysis. The presence/absence of GIs, prophages, and other regions of genome plasticity supported the subdivision of *P. aeruginosa* into two main groups [38,39]. Each group may be drawing upon distinct mobile gene pools at the largest clade grouping level (group 1 vs. group 2). This was in accordance with the core gene-based population structure of *P. aeruginosa*, which contains 4009 core genes within 103 *P. aeruginosa* genomes [40]. However, due to the highly adaptive divergence within the GIs regions, the *P. aeruginosa* pan-genome is continuously extended. Nearly half of the new environmental and industrial isolates had new genotypes, indicating the wide geographically dispersing of the distinct clones [40,41].

**Figure 4:**
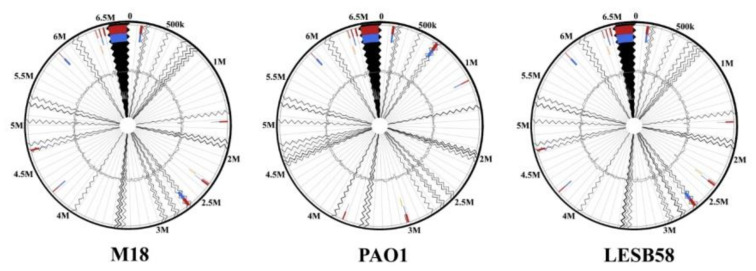
Genomic islands (multicolor) in the *P. aeruginosa* PAEM genome (contigs) against the genomes of reference strains M18, PAO1, and LESB58 analyzed by IslandViewer 4 [38]. Zigzags indicate unidentified sequences between PAEM contigs. Information on the chromosome of *P. aeruginosa* PAEM larger than 6.5 Mb is absent (black zigzag break).

Although *P. aeruginosa* PAEM, similar to the strains DSM 50071^T^, PAO1, and LESB58, is included into the most represented *P. aeruginosa* phylogenetic group based on the whole-genome analysis [39,40], the comparable part of its genome (35 contigs) distinguishes from the DSM 50071^T^ and PAO1 genomes by 10 GIs, containing 122 and 151 CDSs, respectively (Figure 4). Meanwhile, it distinguishes from LESB58 by 11 GIs, containing 160 CDSs, with the longest GI of 34497 bp, according to the analysis by IslandViewer 4 [38].

The *P. aeruginosa* PAEM alignment against the reference genomes of the environmental rizosphere- and rice-derived strains M18 and F9676 resulted in 11 (160 CDSs) and 10 (121 CDSs) GIs, respectively (Figure 4). The largest lengths of the PAEM GIs are 34749 and 28865 bp aligned against the M18 and F9676 genomes, respectively. The locations of PAEM GIs against M18 in comparison with LESB8 are identical (Figure 4). Remarkably, the phylogenetic outlier strain *P. aeruginosa* PA7 [40,41], with the lowest nucleotide sequences similarity (≤90–95%) with PAEM, revealed eight GIs (158 CDSs), with the largest length of 59,689 bp (data not shown). The CDSs of GIs found by IslandViewer 4 coincide with the singletons from the results of the EzBioCloud CG analysis (Table S7).

As the main distinctive traits for *P. aeruginosa* PAEM, the IslandViewer 4 found the GIs containing recombinational DNA repair, replication, and DNA/RNA metabolism systems, such as exodeoxyribonucleases V alpha (RecD), exonucleases SbcCD, RecA/RadA recombinases, superfamily II DNA/RNA helicases (SNF2 family), ribonucleotide reductases (aerobic and anaerobic), error-prone repair homolog of DNA polymerases III, DNA polymerase IV-like protein ImuB, ParA-like proteins; the antibiotic biosynthesis/resistance and iron transport genes encoding colicin/pyosin nuclease family proteins, pyocin S2 immunity protein, ferrichrome–iron receptor, pyoverdine sidechain non-ribosomal peptide synthetases PvdJ; the malonate pathway proteins; T6SS components VgrG proteins and virulence determinants Rhs-family proteins (type VI secretion system), Vap toxin system proteins, cold shock proteins of the CSP family; trans-2,3-dihydro-3-hydroxyanthranilate isomerase (EC 5.3.3.17), conjugative transfer proteins PilL, P, Q, S, U, M in a PFGI-1-like cluster, large exoproteins involved in heme utilization or adhesion, secreted proteins Hcp, thiol peroxidase of Tpx-type, bacteriocin/lantibiotic efflux ABC transporter/ATP-binding proteins, aliphatic amidase AmiE (EC 3.5.1.4), and hypothetical and phage-related proteins. Evidently, the restriction–modification systems are most highly variable among the prokaryotes under an evolutionary drive, since they have themselves been ranked with mobile elements [42].

Nevertheless, many protein sequences, encoded by the GIs regions against PAO1 and LESB58, have been found in PAEM with the high similarity by BLAST in EzBioCloud (data not shown). Some of the PAEM CDSs (absent in PAO1 or/and LESB58) are mainly similar to hypothetical or phage proteins (99–100%) and the functional genes related to the traits of *P. aeruginosa* strains isolated from the hexachlorocyclohexane (chlorinated organic insecticide)-contaminated soil MBT-1 (NCBI ID: CP006853), crude oil IMP66 (NCBI ID: CP028959), rice F9676 (NCBI ID: CP012066.1), tung meal LYT4 (NCBI ID: CP052759), swab from a not healing wound (NCBI ID: CP041785), and mink raising farm PA59 (NCBI ID: NZ_CP024630.1). No sequence similarity at all was found for PAEM_02237 (hypothetical protein) and PAEM_03871 (chromosome partitioning protein ParA) (Tables S1 and S7).

Generally, the results on the PAEM strain are in agreement with the evidence about the low gene variability of *P. aeruginosa* with high levels of their recombination within the species [43,44].

### 2.5. Phylogenetic Analysis

Although most of virulence-, pathogenicity-, and biofilm-associated factors of *P. aeruginosa* PAEM from the frozen meat ready-to cook product showed the higher similarity with the reference genes of PAO1 and LESB58 (Tables S3–S6), one gene, putatively possessing the cold shock protein (Csp) function, has been suggested as a marker gene for the strains adapted to the cold environment. The DNA:DNA hybridization (DDH), which was carried out by the TYGS server (https://tygs.dsmz.de), confirmed the similarity of the PAEM strain to the reference strains with the following percentages: LESB58–96.6%; M18–95.5%; F9676–95.4%; PAO1–95.4%; VET-48–95.2%; and PSE305–94.7% [45].

The genome of the true environmental isolate, such as the biocontrol strain M18 from the sweet melon rhizosphere, is also most similar to that of one of the severe clinical pathogens *P. aeruginosa* strain LESB58 [37]. However, the whole transcriptomic analysis of M18 indicated that 10.6% of the expressed genes are temperature-dependent, with the up-regulation at lower temperature than at 37 °C [37].

Cold shock proteins (Csps) are mainly known to respond to the stabilization of the transcription and translation of proteins to prevent the cell membrane fluidity and enzyme activity decrease due to the temperature downshift (cold shock). Csps may contribute to tolerance against osmotic, oxidative, starvation, pH, and ethanol stress, as well as to host cell invasion for enteropathogens [46]. The CDS for hypothetical protein PAEM_03055 in the cold-adapted *P. aeruginosa* PAEM (Table S1) was predicted as a CspA family protein of stress response and cold shock (beta-ribbon DNA-binding domain), according to the EGGNOG/KEGG/SEED function from the EzBioCloud genome feature data [25].

From all sequenced genomes (4660) from Pseudomonas Genomes DB, 238 BLASTN hits against PAEM_03055 returned [23]. However, the homologues of CspA PAEM_03055 (GeneBank: HZL38_16480) are mostly annotated as “Main chromosome cold shock protein CapB” or “DUF1294 domain-containing protein” with unknown function. The alignment of an amino acid sequence of PAEM_03055 (Diamond BLASTP) resulted in finding 3954 homologues (including resubmitted genome versions) among the complete assemblies of *P. aeruginosa* [23].

**Figure 5:**
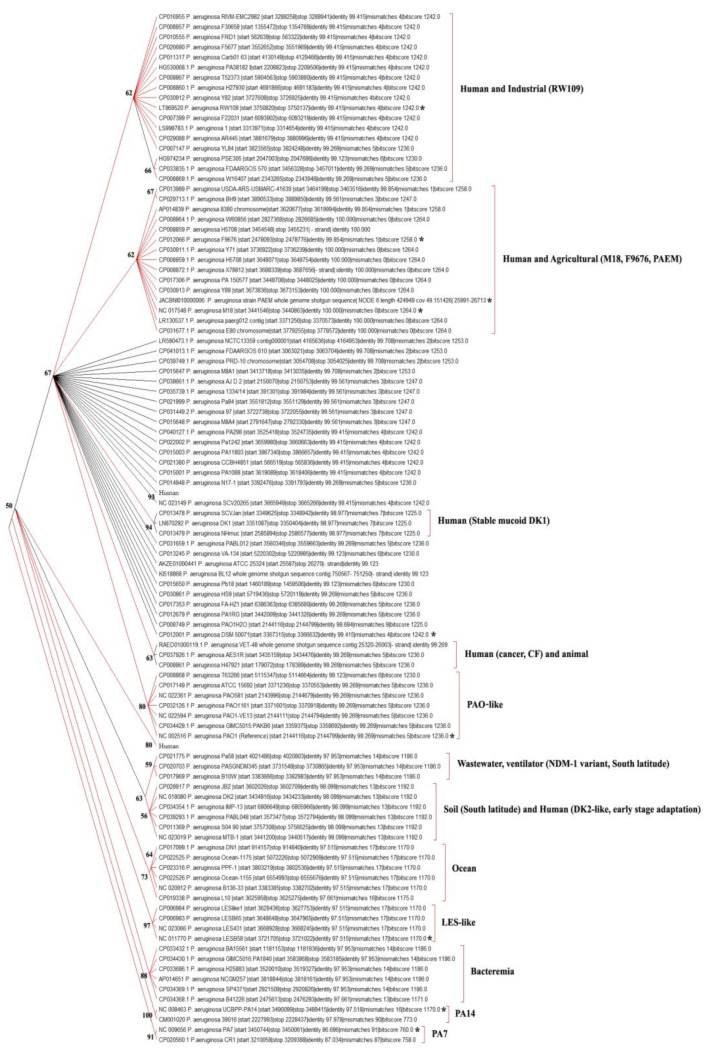
Phylogenetic analysis and evolutionary history for the close homologues of the putative CspA family cold shock protein PAEM_03055 inferred with the Maximum Likelihood method in MEGA X [47]. The most characteristic features of the strains placed into one cluster are indicated behind the squares (host or disease specificity, relation with a reference strain, isolation source, geographic location, etc.).

Phylogenetic analysis of the nucleotide sequences (codons) for the close homologues of PAEM_03055 was conducted in MEGA X [47]. The evolutionary history (evolutionary probability, EP) was inferred using the Maximum Likelihood method and Hasegawa–Kishino–Yano model, plus a discrete Gamma distribution (+G), which due to the lowest BIC scores (Bayesian Information Criterion) were taken to describe the substitution pattern the best [48,49]. The bootstrap consensus tree inferred from 500 replicates [50] is taken to represent the evolutionary history (Figure 5). Branches corresponding to partitions reproduced in less than 50% bootstrap replicates are collapsed. The percentage of replicate trees, in which the associated taxa clustered together in the bootstrap test (100 replicates), is shown next to the branches [50]. Initial trees for the heuristic search were obtained automatically by applying Neighbor-Join and BioNJ algorithms to a matrix of pairwise distances estimated using the Maximum Composite Likelihood (MCL) approach and then selecting the topology with a superior log likelihood value. A discrete Gamma distribution was used to model evolutionary rate differences among sites (five categories (+G, parameter = 0.2394)). This analysis involved 111 nucleotide sequences. Codon positions included were 1st+2nd+3rd+Noncoding [47]. There were in total 728 positions in the final dataset. The CspA sequence of the *P. aeruginosa* strain PA7 was chosen as an outgroup because of the highest bootstrap values in the tree nodes (Figure 5), while the strains clustered similarly independently on outgroups and methods (data not shown).

Although no possibility of inferring a common ancestor of clones for the species *P. aeruginosa*, due to their capability of the fast intraspecific chromosomal recombination [43,44], the cold shock protein CspA has shown an evolutionary probability of the strains origin depending on the sources and geography of their isolation (Figure 5).

The branching pattern of the tree for the strain PAEM and reference agricultural isolates, such as M18 from melon rhizosphere (100% identity) and F9676 from rice (99.85%), is identical. However, the clinical MDR isolates of *P. aeruginosa*, including host-associated 012 (Switzerland, diseased humans); Y71, Y89 (South Korea, sputum of 70-year-old male); E80 (Washington, cystic fibrosis patient); X78812, H5708, W60856, (New York, Memorial Hospital, cancer patients), and PA_150577 (Hong Kong, human) are also with 100% identity of their CspA with that of the agricultural strains and cold-adapted PAEM_03055 (Table S1; GenBank: HZL38_16480).

The strains *P. aeruginosa* 8380 and BH9 from human gut and urine samples, respectively; USDA-ARS-USMARC-41639 from nasopharynx of domestic cow; and NCTC13359 from water bottles are also clustered in the same clade with PAEM, M18, and F9676 (Figure 5). Probably, they all may be highly adapted to negative temperatures, because PAEM withstands long-term storage of at least up to −20 °C in the frozen meat mince containing soy (NCBI BioSample: SAMN15461260). The agricultural soils used for growing plants can also freeze during the winter at least in northern latitudes.

There is the same possibility that the source of the isolate PAEM could be the meat-processing environment or the farm animal itself infected by the soil-derived pseudomonad. The farm animal-associated strain PSE350 is placed in the neighbor clade of the industrial strains, such as the RW109-like strains with the large multireplicon genomes (about 7.0–7.9 million bp) [40].

The farm or agricultural strains N17-1 and HS9 from the soil; AJD2 from the cotton rizosphere; FDAARGOS, NCTC 13359, and PRD10 (animal room) from water bottles, as well as the strains M8A1, M8A4, and Pb18 isolated from the crude oil; and FA-HZ1 and PA1RG from wastewater seem to originate by parallel evolution from the same ancestor as the strains from two clusters of the PAEM- and industrial RW109-related strains (Figure 5). Thus, the representatives of these phylogenetic branches, inferred from the common ancestor, including human-associated strains and the reference strains PAO1 and DSM 50071^T^, may be originated from the environment, particularly the agricultural soils and plants, which, in turn, may be contaminated by the industrial waste. Probably, all of them can be adapted to negative temperatures to varying degrees and other negative environmental factors for their survival.

However, the topology of the ML tree branches indicates that the *P. aeruginosa* strains, with the Csp related to the cold-adapted strains, could have a probability of secondary origination from the high-temperature tolerant pathogenic strains, such as PA7, UCBPP-PA14, or LES-like strains (Figure 5).

Notably, the isolates from the ocean, clustered with an algae-associated DN1 and L10 from a holobiotic reed, are also close to the earlier common ancestor with the LES-like isolates or the isolates mainly causing bacteremia or associated with the burn pathology, which clustered in the separate branch. The strain DK2 that suggested having the traits for early-stage adaptation to the human organism are included in the neighbor branch (Figure 5). Thus, their ancestors putatively belonged to the strains from southerly latitude or from warm-blooded mammals. This is also indicated by the close location of the environmental strains MBT-1 (soil, India), B10W (wastewater, Honolulu), as well as the strains associated with the community-acquired diarrhea B136-33 (Taiwan) or community-acquired pneumonia PA58 (ventilator-associated, Mexico) and PASGNDM345 (sputum, Singapore) (Figure 5).

The phylogenetic outlier strain PA7 from a non-respiratory patient clustered closely to the branch with the strain UCBPP-PA14 from a human burn patient (Figure 5), which is reference for the strains containing the additional exotoxin-encoding gene *exoU* [41]. Remarkably, the route branch PA7 contains the strain CR1 isolated from a chilli rhizosphere in India (NCBI, BioSample: SAMN06673526) (Figure 5). Therefore, it is not surprising that the *P. aeruginosa* strains, including PAEM, have the genes responsible for the plant growth promotion (or pathogenesis) and biocontrol activity (Tables S1 and S3) [37,40,41].

### 2.6. Gene Expression Analysis after Cultivation of PAEM with the Marine Bacterial Alpha-Galactosidase α-PsGal

**Figure 6:**
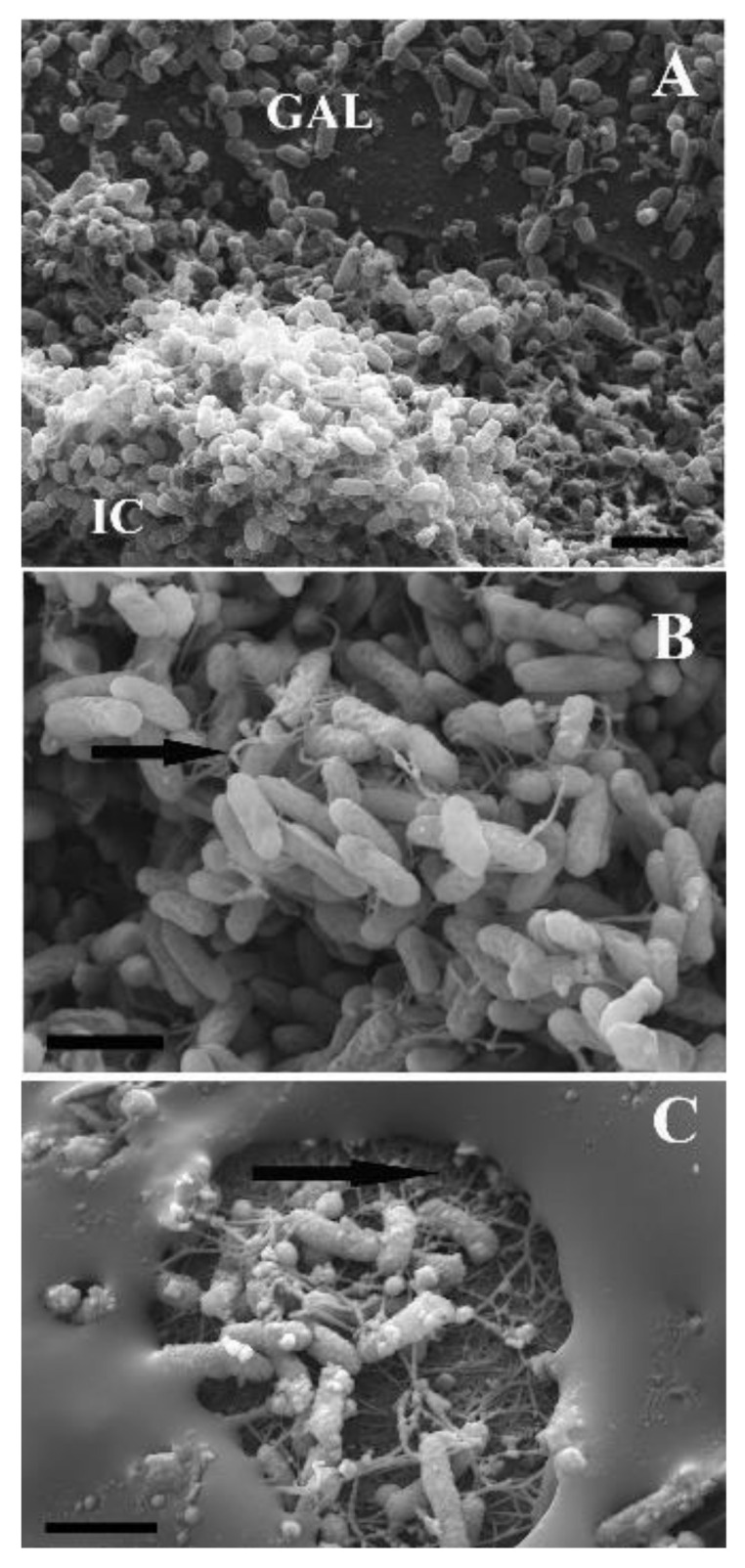
Effect of the marine bacterial galactosidase α-PsGal on the *P. aeruginosa* PAEM mature biofilm (agar Luria-Bertani (LB) medium, 22 °C, 12 h): (**A**) The treated (GAL) and untreated (IC, intact culture) biofilm by the enzyme α-PsGal; (**B**) Intact culture (optically zoomed the IC zone, A), a network of pilus (black arrow) between the bacterial cells; (**C**) An area of the bacterial biofilm treated with the enzyme α-PsGal: the homogenous substance formed by *P. aeruginosa* PAEM in response to the action of the enzyme covers a significant part of the biofilm surface, alternating with the degraded parts of the old biofilm and outer membrane vesicles’ formation on the cells (C, black arrow). The scale bars correspond to 0.5 microns (**A**), 4 microns (**B**), 2 microns (**C**). The method of scanning electron microscopy with the use of microscope Evo 40 (Carl Zeiss) was described earlier [3].

The highly biofilm-forming strain *P. aeruginosa* PAEM has been found to decrease its newly biofilm formation by 60% under supplementation with the cold-active alpha-galactosidase *α-*PsGal from the marine bacterium *Pseudoalteromonas* sp. KMM 701 into the liquid growth medium (Luria-Bertani, LB) at a room temperature without shaking (data not shown). In addition, the mature biofilm destruction was observed as gullies, channels, and deep pits between the colonies, when 15 μL of α-PsGal at a concentration of 0.08 mg/mL (with the activity 1–20 U/mg protein) was applied above the plated *P. aeruginosa* and kept for 12 h at 22 °C (Figure 6A–C). However, the results of electron microscopy, along with the biofilm-degrading effect, revealed a significant change in the morphology of the extracellular matrix after 24 h of incubation with the enzyme α-PsGal (Figure 6). The biofilm changed its structure from being uniformly loose due to the formed voids and channels between the clusters of colonies to an opaque monolithic substance covering a significant part of the cells (Figure 6C). At the same time, the number of the cells decreased, while the remained cells produced putatively outer membrane vesicles (Figure 6C). It is obvious that the *P. aeruginosa* PAEM biofilms under study have galactose-containing substrates for the hydrolysis reaction in the presence of α-PsGal. Probably, the appearance of a large amount of free galactose as a result of the action of the galactosidase could trigger the synthesis of new and/or transformation existing oligo- and polysaccharides in the extracellular matrix by the PAEM cells themselves or with the participation of the transglycosylating activity of α-PsGal [4].

According to the comparative genomics, the PAEM pathogenome, including most of the biofilm-regulating factors, is the same with the reference strains PAO1 and LESB58 (Table S6). Therefore, for evaluating the effect of the cold-active alpha-galactosidase α-PsGal on *P. aeruginosa* PAEM, the levels of gene expression with the established biofilm-regulation functions were determined in the presence of the different concentrations of the enzyme in the cultivation medium (Figure 6, Table 1).

To evaluate the best reference genes for *P. aeruginosa* PAEM, three candidate genes of pyrroline-5-carboxylate reductase (*proC*), sigma factor RpoD (*rpoD*), and chromosomal beta-lactamase (*ampC*) were compared in terms of the expression stability in all experimental and control samples (Table 1). The relative fold change in the expression was evaluated using the RefFinder [61], which integrates different mathematic algorithms (geNorm, Normfinder, BestKeeper and the comparative deltaCt method) to compare and rank the tested candidate reference genes.

**Figure 7:**
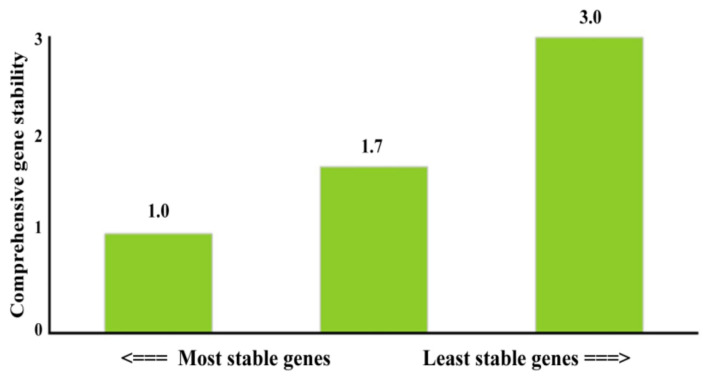
Stability analysis of the candidates for reference genes in *P. aeruginosa* PAEM calculated by RefFinder. Values above the bars indicate geomean of ranking values.

The *proC* and *rpoD* genes showed the most stable expression patterns in all tested experimental sets (Figure 7); thus, this pair was used as an internal control in the relative comparison studies of the biofilm-related genes in *P. aeruginosa* PAEM. Similarly, these genes formed the most stable pair among six candidate housekeeping genes earlier [51].

**Figure 8:**
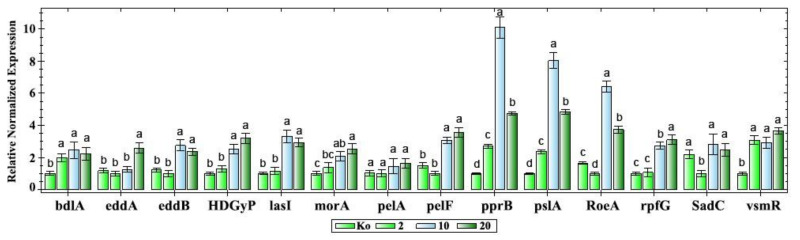
Relative differential expression of the biofilm-related genes in *P. aeruginosa* PAEM, normalized to the geometric mean of two reference genes (evaluated by qPCR), under its cultivation with the a-galactosidase α-PsGal (with the enzymatic activity 2, 10, and 20 units (U/mg per mL) as indicated below diagram). *P. aeruginosa* PAEM grown at the same conditions (50 mL of liquid LB medium, without shaking at 22 °C for 12 h), without an addition of α-PsGal in the nutrition medium LB, was taken as the control (Ko). The names, functions, and references of the genes are in Table 1. Data (mean ± standard error) represent measurements of three independent replicates from three different RNA isolations. Different letters above the bars indicate statistically significant differences of means (*p* < 0.05) for each gene, Fisher’s Least Significant Difference (LSD).

**Figure 9:**
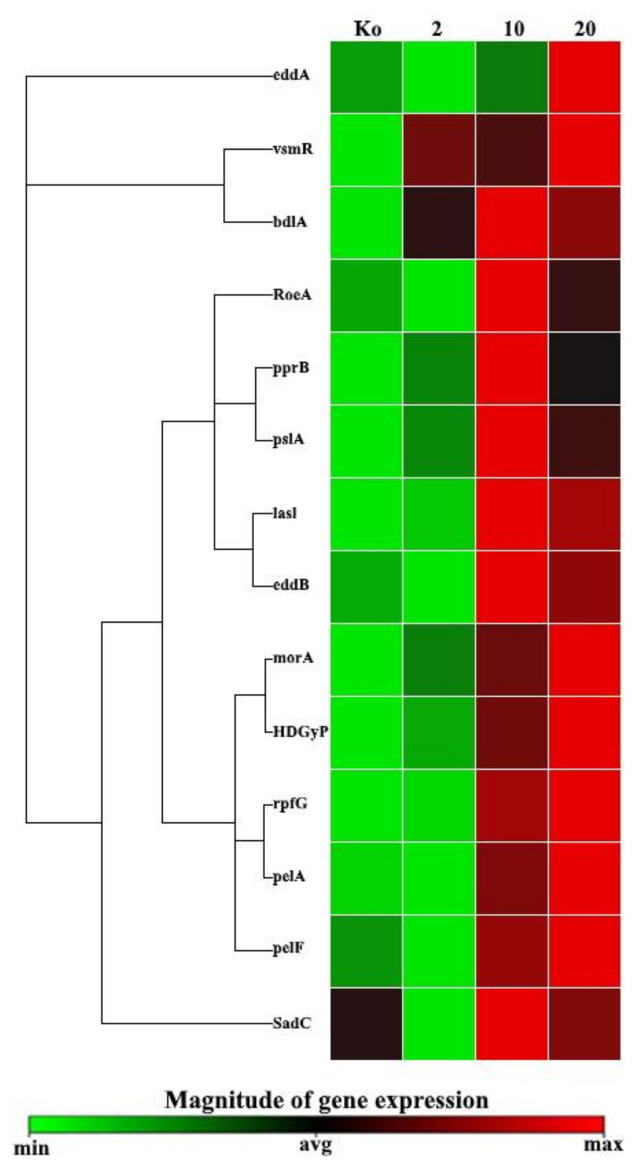
Clustergram and heatmap of the expression data on biofilm-associated genes in the *P. aeruginosa* strain PAEM. Color ranges from green to black and red, according to the magnitude of relative gene expression, as shown in the scale bar. Targets are clustered according to their similarity in the expression pattern.

The expression levels of the biofilm-related genes of *P. aeruginosa* PAEM were evaluated by quantitative PCR (qPCR) (Figure 8). These genes showed the differential expression patterns in both control and experimental conditions under 2, 10, and 20 units of the a-galactosidase α-PsGal supplemented into the growth medium (Figure 8 and Figure 9). Five groups of the genes were identified based on their transcriptional levels.

The first group consists of the genes *bdlA* and *vsmR(rhlR)*, whose expression was increased by 2–3 folds upon the enzyme supplementation, and no dose-dependent effects were observed (Figure 8 and Figure 9). BdlA, a chemotaxis regulator, plays a pivotal role in the dispersion of biofilm, while *vsmR* (*rhlR*), encoding for the regulator RhlR, controls expression of the genes required for biofilm formation, including LasR-specific factors of the QS system in *P. aeruginosa* [8,53,59]. It has been suggested that BdlA may be a link between sensing environmental cues, c-di-GMP levels, and detachment. The *bdl*A mutants were found to have increased adherent properties and intracellular levels of c-di-GMP [59]. Meanwhile, two interconnected acyl-homoserine lactone (acyl-HSL) signal-receptor pairs, 3-oxo-dodecanoyl-HSL-LasR and butanoyl-HSL-RhlR, regulate more than 300 genes, including QS-dependent motility, virulence, and biofilm formation [53].

The second and most abundant group consists of the genes *eddA*, *eddB*, *HDGYP*, *rpfG/C*, *lasl*, *pelF*, and *morA*, whose expression was increased by 1.4-3.5 fold only by 10 and/or 20 units of the enzyme (Figure 8 and Figure 9). This group includes genes participating in QS, extracellular DNA (eDNA) degradation, production of a glucose-rich matrix exopolysaccharide Pel, and flagellar development [53,55,57,58,60].

Many pathogens produce secreted extracellular DNases to avoid an external antimicrobial activity. *P. aeruginosa* encodes an operon of two secreted enzymes, a predicted alkaline phosphatase and DNase. The DNase EddB degrades eDNA to use as a nutrient source. EddA has both alkaline phosphatase and phosphodiesterase activities and protects against antimicrobial activity [60]. Nevertheless, the eDNA degradation may be a factor of the visible changes in the *P. aeruginosa* PAEM biofilm thickness (Figure 6).

HDGYP is a protein domain, which is encoded by the genes, signed here as *HDGYP* and *rpfG/C* (Table 1, Figure 8 and Figure 9), is involved in the hydrolysis of the bacterial second messenger cyclic-di-GMP [58]. Overexpression of these domain-containing proteins was found to inhibit biofilm formation [58].

PelF, a putative glycosyltransferase, encoded by a seven-gene cluster *pel*, is involved in the polysaccharide Pel biogenesis [57].

The absence of regulator motility MorA in the *P. aeruginosa* mutants led to a reduction in biofilm formation, but their motility was unaffected [53]. In addition, the overexpression of flagellin resulted in the decreased adhesion; therefore, it has been hypothesized that flagella might be strongly regulated by MorA at certain stages of bacterial adhesion and biofilm formation [53].

The *lasI* gene was found to be down-regulated in biofilm [55]; therefore, an increase in the level of its expression may coincide with the biofilm degradation/dispersion of *P. aeruginosa* PAEM (Figure 6).

The third group consists of the genes *pslA* and *pprB*, whose expression was extremely activated in a dose-dependent manner from 2 to 10 units of the enzyme, while 20 units of the enzyme slightly decreased this effect, remaining a magnitude higher compared to the control (Figure 8 and Figure 9). The permeability regulator encoded by *pprB* serves as a receiver in a “PprAB” system, which switches bacteria from their free-living cells to multi-cellular biofilm. It was shown that *ppr*B activation is also associated with the increased susceptibility to antibiotics and reduced virulence [56]

Meanwhile, PslA is the first and most important protein necessary for the synthesis of the biofilm matrix polysaccharide Psl [8,12,52,54]. The *P. aeruginosa* polysaccharide Psl could be a galactose- and mannose-rich exopolysaccharide [54]. However, the galactose-containing matrix component is still unknown, which is probably due to the strain-dependent polysaccharide structure specificity [62].

Notably, the nanomolar concentrations of the own *P. aeruginosa* glycoside hydrolases PslG and PelA, belonging to the different metabolic pathways and participating in vivo exopolysaccharide processing, also effectively degraded polysaccharide components of the biofilm matrix, preventing newly biofilms and rapidly disrupted mature biofilms in vitro [63]. The treatments by PelA and PslG were effective against clinical and environmental *P. aeruginosa* isolates and reduced biofilm biomass by 58–94% [63].

The fourth PAEM expression pattern group consists of the genes *roeA* and *sadC*, whose expression was down-regulated by two units of the a-galactosidase α-PsGal, but it was activated by 10 and 20 units of the enzyme (Figure 8 and Figure 9). These genes also participate in the production of extracellular polysaccharides (EPS), biofilm formation, and swarming motility [13,14]. The digyanilate cyclases RoeA and SadC make distinct contributions to the biofilm formation, controlling polysaccharide production and flagellar motility, respectively, with no correlation between levels of c-di-GMP and the observed phenotypic traits with regard to the EPS production and swarming motility [13].

It also can be noted that genes from the third and fourth clustered groups, such as *pprB*, *pslA,* and *roeA*, showed the highest level of up-regulation in response to the a-galactosidase α-PsGal application. At the dose of 10 units, their expression levels were induced 6.4–10.1 fold compared to the untreated cells (Figure 8).

The last fifth cluster group includes one gene *pelA*, whose expression was not altered by α-PsGal (Figure 8 and Figure 9). PelA is involved in the polysaccharide Pel production and important for maintaining of cell-to-cell communication. However, loss of the Pel biosynthesis in the PAO1 strain resulted in no difference in attachment or biofilm development [57]. Thus, the PAEM glucose-containing Pel is not absolutely affected by the presence of the galactosidase and/or free galactose (Figure 8 and Figure 9), and it is also not critical for the biofilm maintenance [57].

The heatmap shown in Figure 9 partly confirmed clustering of the commonly regulated *P. aeruginosa* PAEM biofilm-associated genes. However, as soon as the clustergram shows the data in a hierarchy, based on the degree of similarity of the expression for different targets and samples not considering statistical confidence, it does not fully reflect the observed biological effect.

## 3. Materials and Methods

### 3.1. Isolation and Cultivation of P. aeruginosa PAEM

The *P. aeruginosa* PAEM isolate, with a high biofilm production property, was isolated from a frozen ready-to-cook product, such as cabbage rolls, containing pork, poultry, and soybeans, with a shelf life at 18 °C for up to three months. The strain identification to the species level and cultivation on the appropriate strain-specific nutrient medium were carried out according to the accredited laboratory manuals (ISO/IEC 17025) [3]. The *P. aeruginosa* PAEM biofilm growth, morphology, and antibiofilm activity of the cold-active marine bacterial enzymes were studied by the methods described earlier [3].

### 3.2. Sequencing, Assembly, and Analysis of Genome Sequence data

Whole genome sequencing (WGS) of bacterial isolate was performed using the high-performance next-generation sequencing (NGS) method on a MiSeq Illumina sequencer (Illumina, Inc., San Diego, CA, USA). Chromosomal DNA was isolated using the PureLinkTM Microbiome DNA Purification Kit (Thermo Fisher Scientific) from a pure culture grown on an agar medium. DNA samples were analyzed by agarose gel electrophoresis and Qubit 4 fluorimeter (Invitrogen). DNA fragmentation was performed using Covaris ultrasound equipment (Thermo Fisher Scientific). To prepare the libraries, 1 ng of DNA and a Nextera DNA Flex Library Prep (24 Samples) reagent kit (Illumina, Inc., San Diego, CA, USA) were used. The resulting libraries were sequenced using the MiSeq Reagent Kit v2 (300 cycle) kit (Illumina, Inc., San Diego, CA, USA) for cluster generation and sequencing with a fragment reading length of 250 x 2 base pairs and a productivity of at least 300 Mb. Sequencing quality was monitored by the automated sequencing analysis viewer (Illumina, Inc., San Diego, CA, USA). The overlapping fragments of the input DNA sample (reads) were read with a minimum coating number of 60×. The quality of reads was evaluated using the FastQC program (bioinformatics.com). The processing (trimming) of readings to remove low-quality nucleotides (<Q20) was performed by the Trimmomatic program [64]. The assembly of the cleaned reads into contigs was performed using SPAdes [65]. The quality of assembly of the obtained reads into contigs (quality of the genomic assembly) was assessed using the QUAST program (quality assessment tool for genome assemblies) [66]. The genome contigs were annotated using the RAST (Rapid Annotation using Subsystem Technology) and EzBiocloud (ezbiocloud.net) interactive servers [24,25]. Comparative analyses of the nucleotide sequences of the genes and genomes were also performed using the BLAST, ClustalW, MEGA programs, and the reference sequences from the NCBI, PubMLST, Pseudomonas DB, VFPB (Virulence Factors of Pathogenic Bacteria; http://www.mgc.ac.cn/VFs/), and TYGS (Type (strain) genome server; https://tygs.dsmz.de) websites [23,30,32,45].

### 3.3. Cultivation of P. aeruginosa PAEM in the Presence of α-Galactosidase α-PsGal

To study the effect of the bacterial α-galactosidase on the biofilm-associated genes expression, the *P. aeruginosa* strain PAEM was cultivated in the presence of the recombinant enzyme α-PsGal from the cold-adapted marine bacterium *Pseudoalteromonas* sp. KMM 701. The isolation, purification, and properties of the recombinant α-galactosidase α-PsGal were described earlier [4].

The overnight culture of *P. aeruginosa* PAEM was inoculated in 20 mL of LB medium (pH 7.4) and cultivated at 37 °C in the dark without a shaker for 12 h in the following options: (1) without the addition of the enzyme α-PsGal (control culture); (2) with the addition of α-PsGal with the total activity of 2 units (7.08 μL); (3) with the addition of α-PsGal with the total activity of 10 units (13.1 μL); (4) with the addition of α-PsGal with the total activity of 20 units (18.0 μL).

### 3.4. RNA Isolation and First Strand cDNA Synthesis

The total RNA was isolated with the use of ExtractRNA kit (Evrogen, Moscow, Russia). To evaluate the integrity and purity of RNA, samples were analyzed by using microcapillary electrophoresis chips (Experion, Bio-Rad Laboratories, Inc., Hercules, CA, USA) as described by Veremeichik et al. [67]. Following the protocol of “Evrogen” (Russia), reverse transcription was accomplished to generate the first strand of complementary DNA (cDNA), starting from 1 µg of total RNA.

### 3.5. Real-Time PCR

The gene expression was studied in the enzymatically treated and untreated bacterial samples by using quantitative real-time PCR (qPCR). A CFX96 (Bio-Rad Laboratories, USA) was used with qPCRmix-HS SYBR (Evrogen, Moscow, Russia). Reaction and temperature conditions for the qPCR were described earlier [67]. The gene-specific primer pairs used in the qPCR are listed in Table 1. The primer pairs efficiency of >95% was verified with the standard curves established using serial dilutions of the corresponding PCR products. The primer specificity was checked by BLASTn search and confirmed in qPCR by melting curve analysis. A set of three potential reference genes (*proC*, *rpoD*, and *ampC*) were assessed using RefFinder in the experimental samples and a pair of the best reference genes were selected for further analysis [61]. This study showed that *proC* and *rpoD* form the most stable pair in the tested experimental set.

Data were analyzed using CFX Manager Software (Version 1.5; Bio-Rad Laboratories, Inc., Hercules, CA, USA). Relative expression levels were calculated with the _ΔΔ_Ct method [68] using CFX Manager Software (Version 3.1; Bio-Rad Laboratories). Heat maps were generated using the same Bio-Rad software. Each data point represents the expression level of one target sequence in one sample, relative to the average of the reference genes. Data points are scaled by target and clustered by the degree of similarity of expression. The overall analysis involved three biological replicates derived from three separate RNA extractions, with three technical replicates being analyzed for each of the three biological replicates.

### 3.6. Statistical Analysis

Statistical analysis was achieved using Statistica 10.0 (StatSoft, Inc., Tulsa, OK, USA) with the level of statistical significance being regarded as *p* < 0.05. Comparisons among multiple groups were achieved by ANOVA followed by a multiple comparison protocol. The inter-group comparison was made using Fisher’s protected least significant difference (PLSD) post-hoc test.

## 4. Conclusions

The *P. aeruginosa* strain PAEM, isolated from the environment of meat processing during the ready-to-cook product storage at −20 °C, has a genome typical for this species, with varying degrees of the identity for the coding sequences of the free-living and clinical strains. Although the highest similarity of the PAEM whole genome is with the epidemic strain LESB58 (96.6%), comparative genomics showed that its closest homologues are the soil microorganisms associated with agricultural crops, such as the strains M18 (95.5%) and F7696 (95, 4%). Moreover, the M18 genome itself is also closest to the LESB58 genome. The relationship of PAEM with the agricultural strains is confirmed by the presence of the specific PAEM sequences (singletones) responsible for the synthesis of a wide range of antibiotics for biocontrol, some plant growth factors, and proteins for the utilization of hydrocarbons (by similarity according to the EzBioCloud annotation). Analysis of the PAEM genomic islands (GIs) showed an identical pattern of their location on the M18 and LESB58 chromosomes. In addition, the PAEM singleton encoding for the putative cold shock protein of CspA family (by EzBioCloud) seems to cluster the strains together according to their adaptation, particularly to the soil lifestyle and temperatures, indicating the common ancestor. The protein CspA can potentially be used to analyze the parent clones for the *P. aeruginosa* strains, which are prone to the highest level of chromosome recombination.

Taking into account the adaptation to cold lifestyle, the PAEM strain was treated with the marine bacterial glycoside hydrolase α-PsGal with the pronounced effect of the biofilm growth prevention and degradation. The differential expression patterns of the PAEM biofilm-associated genes, under the growth conditions with supplementation of 2, 10, and 20 units of the enzyme, have reveled their up-regulation (excluding *pelA*) and clusterization based on their transcriptional levels: (1) *bdlA* and *vsmR(rhlR); (*2) *eddA*, *eddB*, *HDGYP*, *rpfG/C*, *lasl*, *pelF*, and *morA;* (3) *pslA* and *pprB; (*4) *roeA* and *sadC;* and (*5*) *pelA.* The genes *pprB*, *pslA,* and *roeA* showed the 6.4–10.1-fold higher level of up-regulation compared to the control in response to the a-galactosidase α-PsGal application at the dose of 10 units. This indicates an increase of the cell permeability mediated by the protein PprB regulation and activation of the glycosyl transferase PslA and diguanylate cyclase RoeA controlling polysaccharide production. Such an active response of the *P. aeruginosa* PAEM cells to the presence of galactose-specific hydrolase may indicate both the presence of a galactose-containing component in the structure of the strain-specific biofilm and the participation of its cleavage products in quorum sensing. However, for the development of the methods for controlling *P. aeruginosa* biofilms associated with cold adaptation, a lot of work will be required to establish the mechanism of the dose-dependent effect of the enzyme, and its concentration and incubation time for the destructive action on the bacterial biofilms.

## Figures and Tables

**Table 1:** Oligonucleotides for the biofilm-associated gene expression analysis by real-time PCR.

Name	Sequence	Target ^1^	Reference ^2^
*pro*C-F *pro*C-R	5′-caggccgggcagttgctgtc-3′ 5′-ggtcaggcgcgaggctgtct-3′	pyrroline-5-carboxylate reductase ProC; PAEM_00339	PA0393 [51]
*amp*R-F *amp*R-R	5′-gcgccatcccttcatcg-3′ 5′-gatgtcgacgcggttgttg-3′	HTH-type transcriptional activator AmpR, PAEM_00821	PA4109 [52]
*mor*A-F *mor*A-R	5′-attccggtgctggaggagatc-3′ 5′-gtcagttccagctccaggcat-3′	motility (flagella) membrane-localized regulator, PAEM_04739	PA4601 [53]
*psl*A-F *psl*A-R	5′-aagatcaagaaacgcgtggaat-3′ 5′-tgtagaggtcgaaccacaccg-3′	UDP-glucose: undecaprenyl-phosphate glucose-1-phosphate transferase, PAEM_02757	PA2231 [12,52,54]
*las*I-F *las*I-R	5′-gcccctacatgctgaagaaca-3′ 5′-cgagcaaggcgcttcct-3′	acyl-homoserine-lactone synthase (LasI,LuxI), PAEM_03681	PA1432 [9,10,55]
*vsm*R*(rhlR)*-F *vsm*R*(rhlR)*-R	5′-tgttcgccgtcctggaa-3 5′-cgccataggcgtagtaatcga-3′	regulatory protein RhlR (VsmR), PAEM_01443	PA3477 [53,55]
*ppr*B-F *ppr*B-R	5′-cggcaacggcaggttct-3′ 5′-catggcctcgatcacttcct-3′	putative two-component system response regulator, LuxR family transcriptional regulator, PAEM_04365	PA4296 [56]
DGC(*roe*A)_F DGC(*roe*A)_R	5′-catggcgcaggcgaaac-3′ 5′-ggaaatcatgtacgccca-3′	diguanylate cyclase (RoeA), PAEM_03920	PA1107 [13,14]
DGC(*sad*C)_F DGC(*sadC*)_R	5′-tcaagcggatcaacgacc-3′ 5′-caccgaaaggctcagggta-3′	diguanylate cyclase (SadC), PAEM_04402	PA4332 [13,14]
*pel*A-F *pel*A-R	5′-aaccaagcctggaacagc-3′ 5′-gcacttcgaaaccgcgat-3′	polysaccharide biosynthesis protein PelA, PAEM_01866	PA3064 [57]
*pel*F*-*F *pel*F*-*R	5′-tggaaaccgcctggagttc-3′ 5′-ggtggagatcgagtgcagcat-3′	polysaccharide biosynthesis protein PelF, PAEM_01871	PA3059 [57]
*rpf*G/C*-*F *rpf*G/C_R	5′-gctcgatgtgaacatgcc-3′ 5′-cattggccttcaattgca-3′	two-component system response regulator, cyclic di-GMP phosphodiesterase, PAEM_04964	PA4781 [58]
*HDGYP*_F *HDGYP*_R	5′-tggatcgatgccagcaag-3′ 5′-cggagatttcgtccacca-3′	metal dependent phosphohydrolase, cyclic di-GMP phosphodiesterase, PAEM_00823	PA4108 [58]
*bdl*A-F *bdl*A-R	5′-atcatgtcggcgatctcc-3′ 5′-gctgaacatcggcgaact-3′	chemotaxis regulator, biofilm dispersion protein BdlA, PAEM_03690	PA1423 [59]
*edd*A-F *edd*A-R	5′-tgtcgaggacgaacacgtc-3′ 5′-gaccacgagaccaccaaca-3′	extracelullar DNA degradation protein, EddA, PAEM_01021	PA3910 [60]

**^1^** The targeted genes’ IDs and annotation are from the Table S1; **^2^** The reference genes’ IDs are from the whole genome sequence of the reference strain *P. aeruginosa* PAO1 (GCF_000006765.1).

## Supplementary Materials

The following are available online at https://www.mdpi.com/1422-0067/21/20/7666/s1, Table S1: Coding DNA sequences (CDS) of *Pseudomonas aeruginosa* PAEM according to EzBioCloud genome annotation, Table S2: Coding DNA sequences (CDS) from the *P. aeruginosa* strain PAEM related to the resistance functions (by similarity in EzBioCloud), Table S3: Antibiotic biosynthesis genes in the *P. aeruginosa* strain PAEM, Table S4: Quorum sensing (QS)-related genes of *P. aeruginosa* strain PAEM (by EzBioCloud sequence similarity), Table S5: Biofilm and exopolysaccharide regulation genes of the *P. aeruginosa* strain PAEM (by EzBioCloud database similarity), Table S6: Comparative pathogenomics of Pseudomonas (based on the Table saved from VFDB (http://www.mgc.ac.cn/VFs/), Table S7: Comparative genomics (EzBioCloud CG).
