# Supplementary material for: Genomic Features of a Food-Derived Pseudomonas aeruginosa Strain PAEM and Biofilm-Associated Gene Expression under a Marine Bacterial α-Galactosidase
Source: Int J Mol Sci. 2020 Oct 16;21(20):7666. doi: 10.3390/ijms21207666 (PMC7593944; doi:10.3390/ijms21207666)
Supplement: Supplementary file 1 [file ijms-21-07666-s001.zip › Table S4_QS_genes.docx]

**Table S4.** Quorum sensing (QS)-related genes of *P. aeruginosa* strain PAEM (by EzBioCloud sequence similarity).

| **CDS Products** | **Function** | **CDS ID Table S1** |
| --- | --- | --- |
| CAI-1 autoinducer sensor kinase/phosphatase CqsS | Senses the quorum-sensing autoinducer CAI-1 ((S)-3- hydroxytridecan-4-one) which probably functions as an intragenus signal. The sensory signal is then relayed to LuxU and LuxO | 00097 |
| Quorum-quenching N-acyl-homoserine lactonase | Probable hydrolase inhibiting the signaling pathway for the streptomycin production and aerial mycelium growth in S.griseus. Thus, serves as a defensive strategy against competing bacteria. Belongs to the metallo-beta-lactamase superfamily. | 00729 |
| Regulatory proteins (activator RhlR and repressor QscR) | Transcriptional activation of the rhlAB genes encoding the rhamnosyltransferase, as well as of elastase structural gene (lasB). Binds to autoinducer molecules BHL (N-butanoyl-L-homoserine lactone), and HHL (N-hexanoyl-L-homoserine lactone). Belongs to the autoinducer-regulated transcriptional regulatory protein family; Contains 1 HTH luxR-type DNA-binding domain. P.aeruginosa QscR quorum-sensing control repressor (03113). | 01443, 3113 |
| Acyl-homoserine-lactone synthase | Required for the synthesis of BHL (N-butanoyl-L- homoserine lactone), and HHL (N-hexanoyl-L-homoserine lactone) autoinducer molecules which bind to RhlR and thus acts in elastase biosynthesis regulation. Belongs to the autoinducer synthase family.; KEGG: pae:PA3476 acyl homoserine lactone synthase | 01444 |
| Acyl-homoserine-lactone synthase (lasI, [autoinducer synthesis protein LasI](http://www.pseudomonas.com/feature/show?id=105632)) with transferase activity molecular function | Required for the synthesis of PAI consisting of 3-oxo-N- (tetrahydro-2-oxo-3-furanyl)-dodecanamide also known as N-(3- oxododecanoyl)homoserine lactone, an autoinducer molecule, which binds to LasR and thus acts in elastase biosynthesis regulation. Belongs to the autoinducer synthase family.; KEGG: pae:PA1432 acyl homoserine lactone synthase | 03681 |
| HTH-type transcriptional regulator VqsM | Transcriptional regulator involved in both the repression (at least 99 genes, such as mexR and algU) and in the activation (at least 203 genes, such as mvfR, rsaL, vqsR and rpoS) of regulatory or putative regulatory proteins, which are implicated in quorum sensing, virulence and multidrug resistance; Contains 1 HTH araC/xylS-type DNA-binding domain. | 01835, 02384, 05570 |
| S-methyl-5'-thioinosine phosphorylase | Catalyzes the reversible phosphorylation of S-methyl-5'- thioinosine (MTI) to hypoxanthine and 5-methylthioribose-1- phosphate. Involved in the breakdown of S-methyl-5'-thioadenosine (MTA), a major by-product of polyamine biosynthesis. Catabolism of (MTA) occurs via deamination to MTI and phosphorolysis to hypoxanthine. Involved in quorum sensing; Belongs to the PNP/MTAP phosphorylase family. MTAP subfamily.; KEGG: pae:PA3004 5'-methylthioinosine phosphorylase | 01926 |
| 2-heptyl-3-hydroxy-4(1H)-quinolone synthase | Involved in the terminal step of the biosynthesis of quinolone which in addition to serve as a potent signal for quorum sensing, chelates iron and promotes the formation of membrane vesicles (MVs). Catalyzes the hydroxylation of 2-heptyl-4- quinolone (C7-HHQ) to yield 2-heptyl-3-hydroxy-4-quinolone (PQS). PqsH is also able to hydroxylate HHQ analogs having alkyl side- chain lengths of 3 (C3-HHQ), 5 (C5-HHQ) and 9 (C9-HHQ) carbons with low catalytic efficiencies; Belongs to the 3-hydroxybenzoate 6-hydroxylase family.; KEGG: pae:PA2587 2-heptyl-3-hydroxy-4(1H)-quinolone synthase | 02385 |
| Acyl-homoserine-lactone acylase | Catalyzes the deacylation of acyl-homoserine lactone (AHL or acyl-HSL), releasing homoserine lactone (HSL) and the corresponding fatty acid. Possesses a specificity for the degradation of long-chain acyl-HSLs (side chains of 11 to 14 carbons in length). Degrades 3-oxo-C12-HSL, one of the two main AHL signal molecules of P.aeruginosa, and thereby functions as a quorum quencher, inhibiting the las quorum-sensing system. Therefore, may enable P.aeruginosa to modulate its own quorum- sensing-dependent pathogenic potential. Also appears to be required for pyoverdin biosynthesis; Belongs to the peptidase S45 family.; KEGG: pae:PA2385 and PA1032 acyl-homoserine-lactone acylase | 02601, 03996 |
| Transcriptional activator protein LasR | Activation of elastase LasB gene. Binds to the PAI autoinducer. Belongs to the autoinducer-regulated transcriptional regulatory protein family; Contains 1 HTH luxR-type DNA-binding domain. | 03683 |
| Transcriptional activator protein BjaR1 | Response to the quorum-sensing autoinducer IV-HSL (isovaleryl-homoserine lactone). Activates BjaI expression. Is sensitive to IV-HSL at concentrations as low as 10 pM; Belongs to the autoinducer-regulated transcriptional regulatory protein family; Contains 1 HTH luxR-type DNA-binding domain. | 03889 |
| Transcriptional regulatory protein PmpR | Regulation of the quinolone signal (PQS) system and of pyocyanine production. Negatively regulates the quorum-sensing response regulator pqsR of the PQS system by binding to its promoter region; Belongs to the TACO1 family. | 04057 |
