# Supplementary material for: Genomic Features of a Food-Derived Pseudomonas aeruginosa Strain PAEM and Biofilm-Associated Gene Expression under a Marine Bacterial α-Galactosidase
Source: Int J Mol Sci. 2020 Oct 16;21(20):7666. doi: 10.3390/ijms21207666 (PMC7593944; doi:10.3390/ijms21207666)
Supplement: Supplementary file 1 [file ijms-21-07666-s001.zip › Table S5_EPS_genes.docx]

**Table S5.** Biofilm and exopolysaccharide regulation genes of *P. aeruginosa* strain PAEM (by EzBioCloud).

| **CDS Product** | **Function** | **CDS ID from**  **Table S1** | **References** |
| --- | --- | --- | --- |
| D-arginine dehydrogenase, DauA | Is highly expressed within the cystic fibrosis (CF) lung, and it is required for virulence via the optimal production of hydrogen cyanide, pyocyanine, pyoverdine, rhamnolipid and alginate during biofilm formation (PubMed:24011342). Involved in the catabolism of D-lysine and D-arginine. Under aerobic conditions, the arginine succinyltransferase (AST) and arginine transaminase (ATA) pathways are 2 major routes for L-arginine utilization as the sole source of carbon and nitrogen. The D-to-L racemization of arginine by DauA and DauB is necessary, before to be channeled into the AST and/or ATA pathways. DauA catalyzes the flavin-dependent oxidative deamination of D-arginine into 2- ketoarginine (2-KA) and ammonia (PubMed:3141581, PubMed:19139398, PubMed:19850617, PubMed:20809650). It has also dehydrogenase activity towards D-lysine, D-tyrosine, D-methionine, D- phenylalanine, D-ornithine, D-histidine and D-leucine as substrates (PubMed:19850617, PubMed:20809650); Belongs to the FAD-dependent glycerol-3-phosphate dehydrogenase family | 01066 | KEGG: dji:CH75_22885, D-arginine dehydrogenase;  SEED: tress Response, Osmotic stress, Choline_and_Betaine_Uptake_and_Betaine_Biosynthesis,Sarcosine oxidase beta subunit (EC 1.5.3.1) |
| Probable biofilm formation methyltransferase WspC | Involved in biofilm formation; Contains 1 cheR-type methyltransferase domain; Contains 1 TPR repeat | 01232 | KEGG: chemotaxis protein methyltransferase WspC;  SEED: motility and chemotaxis protein methyltransferase CheR (EC 2.1.1.80) |
| Alginate biosynthesis proteins AlgF, AlgJ, AlgX | Together with AlgI and AlgJ, forms an inner membrane complex, which probably interacts with the alginate polymerization- transport complex and adds acetyl groups at the O-2 and O-3 positions of mannuronate residues. Acetylation of alginate is important for the architecture of biofilms and increases resistance to opsonic killing in the host.  AlgX protects alginate from degradation as the polymer traverses the periplasm, and plays a role in its O-acetylation. Acetylation of alginate causes the cells in the biofilm to adhere better to lung epithelium, form microcolonies, and resist the effects of the host immune system and/or antibiotics. | 01391, 01392, 01395 | KEGG: alginate O-acetyltransferase complex proteins AlgF, AlgJ, AlgX;  SEED: Cell Wall and Capsule, Capsular and extracellular polysaccharides, Alginate_metabolism, alginate o-acetyltransferase AlgF, AlgJ, AlgX |
| dTDP-glucose 4,6-dehydratase | Involved in biofilm formation; Belongs to the polysaccharide synthase family | 01783 | KEGG: cgb:cg0417 dTDP-glucose 4,6-dehydratase;  SEED: CBSS-296591.1.peg.2330, Nucleoside-diphosphate sugar epimerase/dehydratase |
| Sporulation kinase D | Phosphorylates the sporulation-regulatory protein spo0F and, to a minor extent, is responsible for heterogeneous expression of spo0A during logarithmical growth. Also phosphorylates spo0A under biofilm growth conditions; Contains 1 histidine kinase domain. | 02051 | KEGG: polar amino acid transport system substrate-binding protein;  SEED: Stress Response, SigmaB_stress_responce_regulation, Putative SigmaB asociated two-component system sensor protein |
| CRISPR-associated endonuclease Cas6/Csy4 | Complements a csy4 disruption in *Pseudomonas aeruginosa* PA14, restoring inhibition of biofilm formation and generation of crRNA; Belongs to the CRISPR-associated endoribonuclease Cas6 family. Cas6f/Csy4, subtype I-F/Ypest subfamily.; | 02555 | KEGG: eci:UTI89_C0896, CRISPR-associated endonuclease Csy4; Acting on ester bonds |
| Biofilm dispersion proteins BdlA | Essential for biofilm dispersion by sensing environmental cues. May be involved in sensing and transducing signals within cells, resulting in the modulation of c-di-GMP levels, swimming motility and adhesiveness of the bacterial cell surface; Contains 1 methyl-accepting transducer domain; Contains 2 PAC (PAS-associated C-terminal) domains; Contains 2 PAS (PER-ARNT-SIM) domains. | 03086, 03690 | KEGG: methyl-accepting chemotaxis proteins;  EGGNOG: Histidine kinase |
| Protein-serine/threonine phosphatase | Dephosphorylates PrkC and FusA (elongation factor G). PrpC and prkC are cotranscribed, which suggests that they form a functional couple in vivo, PrpC's primary role being possibly to counter the action of PrkC. May be involved in sporulation and biofilm formation. Does not seem to be involved in stress response; Contains 1 PPM-type phosphatase domain | 03244 | KEGG: azo:azo1289, protein phosphatase |
| Histidine kinase | Member of the two-component regulatory system RpfG/RpfC, which is required for full virulence and for formation and dispersal of biofilms. Involved in sensing and responding to the diffusible signaling factor (DSF), which is essential for cell- cell signaling. RpfC is probably a sensor of environmental signals, including DSF, which is autophosphorylated at a histidine residue in response to the signal. Then, probably activates RpfG via a four-step phosphorelay. May also negatively regulate the production of DSF, independently of RpfG; Contains 1 histidine kinase domain; Contains 1 HPt domain; Contains 1 response regulatory domain | 03727 | KEGG: ttc:FOKN1_2412, two-component system, sensor histidine kinase RpfC |
| Anthraniloyl-CoA anthraniloyltransferase | Biosynthesis of a number of signaling molecules, quinolone signal 2-heptyl-3-hydroxy-4(1H)- quinolone (PQS), 2-heptyl-4-hydroxyquinoline (HHQ) and 2,4- dihydroxyquinoline (DHQ) for normal biofilm formation. The exact reaction mechanism is still under debate. According to PubMed:18728009, a covalent anthraniloyl-PqsD intermediate is formed, which then condenses with malonyl-CoA or malonyl-acyl carrier protein (malonyl-ACP) to form the short-lived intermediate 3-(2-aminophenyl)-3-oxopropanoyl-CoA. An intramolecular rearrangement of this intermediate can give rise to 2,4-dihydroxyquinoline (DHQ) (in vitro). Alternatively (PubMed:21425231), DHQ and HHQ biosynthesis could proceed via a decarboxylative Claisen condensation of a beta-ketoacid and anthraniloyl-CoA; Belongs to the FabH family | 04032 | KEGG: anthraniloyl-CoA anthraniloyltransferase; PAO1: PA0999 |
| HTH-type transcriptional repressor BluR | Controls the expression of several small proteins that may play a role in biofilm maturation. Binds to and represses the operator of the ycgZ-ymgA-ariR-ymgC operon, and regulates ynaK. BluF antagonizes binding upon blue light (470 nm) irradiation. Blue light may increase the affinity of BluF for BluR, allowing it to be released from its operator; Contains 1 HTH merR-type DNA-binding domain. | 04836 | KEGG: MerR family transcriptional regulator, light-induced transcriptional regulator |
| Esterase EstA | Required for rhamnolipid production, all kinds of cell motility (swimming, swarming, and twitching), and biofilm formation; the exact role of EstA in these processes is unclear. In vitro, has pronounced esterase activities towards p-nitrophenyl esters of short acyl chain length (C4-C6) and Tween detergents. Also shows relatively high activity towards beta-naphthyl butyrate, whereas its activities towards triacylglycerols and acyls-CoA are negligible; Belongs to the 'GDSL' lipolytic enzyme family; Contains 1 autotransporter (TC 1.B.12) domain. | 05303 |  |
| Phosphomannomutase | Highly reversible phosphoryltransferase. Produces a precursor for alginate polymerization, the alginate layer causes a mucoid phenotype and provides a protective barrier against host immune defenses and antibiotics. Also involved in core lipopolysaccaride (LPS) biosynthesis due to its phosphoglucomutase activity. Essential for rhamnolipid production, an exoproduct correlated with pathogenicity. Required for biofilm production. The reaction proceeds via 2 processive phosphoryl transferase reaction; first from enzyme-phospho-Ser-108 to the substrate (generating a bisphosphorylated substrate intermediate and a dephosphorylated enzyme), a 180 degree rotation of the intermediate (probably aided by movement of domain 4), and subsequent transfer of phosphate back to the enzyme; Belongs to the phosphohexose mutase family | 05568 | KEGG: phosphomannomutase/ phosphoglucomutase;  PAO1: PA5322 |
| UDP-glucose:undecaprenyl-phosphate glucose-1-phosphate transferase  (polysaccharide biosynthesis protein PslA) | Capsular and extracellular polysaccharides biosynthesis protein (SEED and KEGG function). Rhamnose-containing glycans. Initiating enzyme for the synthesis of the exopolysaccharide xanthan. Catalyzes the transfer of the glucose- 1-phosphate moiety from UDP-Glc onto the carrier lipid undecaprenyl phosphate (C55-P), forming a phosphoanhydride bond yielding to glucosyl-pyrophosphoryl-undecaprenol (Glc-PP-C55); Belongs to the bacterial sugar transferase family. | 02757 | KEGG: polysaccharide biosynthesis protein PslA;  PAO1: PA2231 |
| Diguanylate cyclase, RoeA | Contains 1 EAL domain; Contains 1 GGDEF domain; Contains 1 MHYT domain; Contains 1 PAS (PER-ARNT-SIM) domain. | 03920 | KEGG: diguanylate cyclase; PAO1: PA1107 |
