# Supplementary material for: Genomic Features of a Food-Derived Pseudomonas aeruginosa Strain PAEM and Biofilm-Associated Gene Expression under a Marine Bacterial α-Galactosidase
Source: Int J Mol Sci. 2020 Oct 16;21(20):7666. doi: 10.3390/ijms21207666 (PMC7593944; doi:10.3390/ijms21207666)
Supplement: Supplementary file 1 [file ijms-21-07666-s001.zip › Table_S3_Antibiotic_biosynthesis CDSs.docx]

**Table S3.** Antibiotic biosynthesis genes in the *P. aeruginosa* strain PAEM.

| **CDS Products** | **Functional Annotation**  **(by EzBioCloud)** | **CDS ID**  **from Table S1** | **Similarity with Reference PAO1** |
| --- | --- | --- | --- |
| L-fuculose-phosphate aldolase | May mediate the 2 consecutive oxidative decarboxylation steps in the biosynthesis of the prenylated hydroxybenzoic acid moiety of novobiocin, an aminocoumarin family antibiotic that targets bacterial DNA gyrases. Belongs to the aldolase class II family.; KEGG: pap:PSPA7_0310 L-fuculose-phosphate aldolase | PAEM_00165 (contig 1) | 99.6% probable aldolase PA0224 |
| Transcription regulatory protein PrtN | Activator of bacteriocin biosynthesis | PAEM_00597 (contig 5) | 97.7% PrtN PA0610 |
| Ribonucleoside-diphosphate reductase | Represses the promoter activity of the prtN gene. Contains 1 HTH cro/C1-type DNA-binding domain.; KEGG: sagu:CDO87_13670 ribonucleoside-diphosphate reductase alpha chain | PAEM_00598 (contig 5) | 99.5% transcriptional regulator prtR PA0611 |
| Bacitracin synthase | Induces peptide synthesis, activates and incorporates five amino acids, forms a thiazoline ring between the first two amino acids and incoporates a D-glutamine in the fourth position. Belongs to the ATP-dependent AMP-binding enzyme family; Contains 5 acyl carrier domains. | PAEM_00854 (contig 11) | 99.7% probable nonribosomal peptide synthetase PAO4078 |
| Validamycin A dioxygenase | Removes, in the presence of oxygen, 4 hydrogen atoms from delta-L-(alpha-aminoadipyl)-L-cysteinyl-D-valine (ACV) to form the azetidinone and thiazolidine rings of isopenicillin. Belongs to the iron/ascorbate-dependent oxidoreductase family; Contains 1 Fe2OG dioxygenase domain.; KEGG: shy:SHJG_0280 validamycin A dioxygenase | PA0572_00738 (contig 9) | 99.8% isopenicillin-N synthase PA4191 |
| dTDP-3-amino-3,4,6-trideoxy-alpha-D-glucopyranose | S-adenosyl-L-methionine-dependent methyltransferase involved in the biosynthesis of desosamine, found in certain macrolide antibiotics such as erthyromycin, azithromycin, and clarithromycin. Catalyzes the last step in the biosynthesis of dTDP-desosamine; Belongs to the methyltransferase TylM1/DesVI family.; KEGG: saq:Sare_2049 dTDP-4-amino-2,4-dideoxy-beta-L-xylose N-methyltransferase; Methyltransferases | PAEM_00751 (contig 9) | 99.9% SAM-dependent methyltransferase , EftM PA4178 |
| Aclacinomycin methylesterase | Involved in the biosynthesis of the anthracycline aclacinomycin, which is an aromatic polyketide antibiotic that exhibits high cytotoxicity and is widely applied in the chemotherapy of a variety of cancers. Catalyzes the removal of the methoxy group from the C-15 position of aclacinomycin T and A to yield 15-demethoxyaclacinomycin T and A, respectively; Belongs to the AB hydrolase superfamily. Hydrolase RdmC family.; KEGG: ag:AAA83422 aclacinomycin methylesterase | PAEM_01352 (contig 11) | 99.2 % probable hydrolase PA3586 |
| Cytochrome P450 monooxygenase PikC | Catalyzes the hydroxylation of narbomycin to give rise to pikromycin, and of 10-deoxymethymycin (YC-17) to give rise to methymycin and neomethymycin during macrolide antibiotic biosynthesis. In addition, produces low amounts of neopicromycin, novapikromycin and novamethymycin. Requires the participation of a ferredoxin and a ferredoxin reductase for the transfer of electrons from NADPH to the monooxygenase; Belongs to the cytochrome P450 family. | PAEM_01595 (contig 12) | 99.8% cytochrome P450 PA3331 |
| Mycosubtilin synthase subunit B | This protein is a multifunctional enzyme, able to activate and polymerize the amino acids Tyr, Asn, Gln and Pro as part of the synthesis of mycosubtilin. The Asn and Gln residues are further epimerized to the D-isomer form. The activation sites for these amino acids consist of individual domains. Belongs to the ATP-dependent AMP-binding enzyme family; Contains 4 acyl carrier domains.; KEGG: pae:PA2893 citronellyl-CoA synthetase; Acid--thiol ligases | PAEM_02040 (contig 15) | 99.5% putative very-long chain acyl-CoA synthetase atuH  PA2893 |
| Chloride peroxidase | Chlorinates and brominates suitable organic compounds. Involved in the biosynthesis of the antibiotic pyrrolnitrin. Belongs to the bacterial non-heme bromo- and chloro- peroxidases family.; KEGG: enc:ECL_02122 non-heme chloroperoxidase | PAEM_02255 (contig 15) | 99.5% chloroperoxidase precursor  PA2717 |
| Linear gramicidin dehydrogenase LgrE | In the final step of gramicidin biosynthesis, reduces the pentadecapeptide-aldehyde intermediate, that is released from the terminal module of the non-ribosomal peptide synthetase LgrD, to the final product ethanolamine-containing gramicidin. Belongs to the thioesterase family. | PAEM_02572 (contig 17) | 99.3% probable thioesterase  PA2411 |
| Enterobactin synthase | Activates five amino acids, incorporates two D-amino acids, releases and cyclizes the mature bacitracin. Belongs to the ATP-dependent AMP-binding enzyme family; Contains 5 acyl carrier domains.; KEGG: src:M271_30130 enterobactin synthetase component F | PAEM_02682 (contig 20) | 99.7% ambB PA2305 |
| Dapdiamide synthesis protein DdaC (2 genes) | nvolved in dapdiamide antibiotics biosynthesis. Catalyzes the alpha-ketoglutarate-dependent epoxidation of the covalently bound N-beta-fumaramoyl-DAP-S-DdaD to generate N-beta- epoxysuccinamoyl-DAP in thioester linkage to DdaD. | PAEM_02683 (contig 20)  PAEM_02684 (contig 20) | 99.5% ambC  PA2304  99.7% ambD PA2303 |
| Mannose-1-phosphate guanylyltransferase | Produces a precursor for alginate polymerization. The alginate layer provides a protective barrier against host immune defenses and antibiotics; Belongs to the mannose-6-phosphate isomerase type 2 family.; KEGG: pae:PA2232 mannose-1-phosphate guanylyltransferase / mannose-6-phosphate isomerase | PAEM_02756 (contig 21) | 99.7% pslB PA2232 |
| Tetracenomycin polyketide synthesis O-methyltransferase TcmP | KEGG: sgu:SGLAU_26330 O-methyltransferase; Methyltransferases | PAEM_03155 (contig 26) | 99.3% hypothetical protein PA1860 |
| Puromycin resistance protein pur8 (2 genes) | May be involved in active puromycin efflux energized by a proton-dependent electrochemical gradient. In addition, it could be implicated in secreting N-acetylpuromycin, the last intermediate of the puromycin biosynthesis pathway, to the environment. Belongs to the major facilitator superfamily. EmrB family. | PAEM_03761 (contig 27)  21contig PAEM_02956 (contig 21) | 99.4% and 99.6%  probable major facilitator superfamily (MFS) transporter PA1262 and PA2055 |
| Gramicidin S synthase | This protein is a multifunctional enzyme, able to activate and polymerize the amino acids Pro, Val, Orn and Leu. Activation sites for these AA consist of individual domains. Belongs to the ATP-dependent AMP-binding enzyme family; Contains 4 acyl carrier domains. | PAEM_03803 (contig 27) | 99.7% hypothetical protein PA1221 |
| Caffeoyl-CoA O-methyltransferase | 4-O-methyltransferase for the lactone ring of midecamycin and other macrolide antibiotics. Belongs to the class I-like SAM-binding methyltransferase superfamily. Cation-dependent O- methyltransferase family.; KEGG: mmw:Mmwyl1_2374 caffeoyl-CoA O-methyltransferase | PAEM_03824 (contig 27) | 99.6% conserved hypothetical protein PA1200 |
| nebramycin 5' synthase | TobZ is involved in the biosynthesis of the 2- deoxystreptamine-containing aminoglycoside antibiotics such as nebramycin 5 and 6-O-carbamoylkanamycin. Catalyzes the hydrolysis of carbamoyl phosphate and its subsequent adenylation by ATP to yield O-carbamoyladenylate. Then it catalyzes the transfer of the carbamoyl moiety from O-carbamoyladenylate to the tobramycin 6- hydroxy group to yield nebramycin 5. It catalyzes the same reaction with kanamycin A. These reactions are considerably slower in the presence of deoxy-ATP; Belongs to the NodU/CmcH family.; KEGG: pae:PA5005 carbamoyltransferase; Carboxy- and carbamoyltransferases | PAEM_05197 (contig 32) | 99.7% probable carbamoyl transferase PA5005 |
