# Supplementary material for: Genomic Features of a Food-Derived Pseudomonas aeruginosa Strain PAEM and Biofilm-Associated Gene Expression under a Marine Bacterial α-Galactosidase
Source: Int J Mol Sci. 2020 Oct 16;21(20):7666. doi: 10.3390/ijms21207666 (PMC7593944; doi:10.3390/ijms21207666)
Supplement: Supplementary file 1 [file ijms-21-07666-s001.zip › Table S2.docx]

**Table S2.** Coding DNA sequences (CDSs) from the *P. aeruginosa* strain PAEM related to the resistance functions (by similarity in EzBioCloud).

| **CDS Products** | **Number of Genes** | **Resistance** | **CDS ID from Table S1** |
| --- | --- | --- | --- |
| penicillin amidases, peptidases, peptidoglycan glycosyltransferase (penicillin-binding proteins) | 6 | Penicillin | [00245](javascript:void(0)), [00929](javascript:void(0)); [03118](javascript:void(0)), [04490](javascript:void(0)); [04880](javascript:void(0)), [05237](javascript:void(0)) |
| kanamycin kinase | 1 | kanamycin, neomycin, paromomycin, ribostamycin, butirosin, gentamicin B | 00811 |
| dihydrofolate reductase | 1 | Trimethoprim, methotrexate | 00294 |
| 2-nitroimidazole transporters | 2 | Azomycin | 00213, 03403 |
| swarming motility protein SwrC; lipid A phosphoethanolamine transferase; multidrugs and acriflavin resistance proteins (4 genes) | 7 | Surfactin, antimicrobial lipopeptide surfactants, puromycin, tetraphenylarsonium chloride, ethidium bromide and methyl viologen | 00077; 06616, 03042; 01566, 02097, 03524, 05181 |
| fusaric acid resistance proteins FusA (2 genes) and FusB (2 genes) | 4 | Mycotoxin | 00721, 01037; 01038, 04152 |
| bicyclomycin resistance protein | 1 | Sulfonamide | 00794 |
| Chloramphenicol (3 genes) and antibiotic transporters ArpB (2 genes); ArpC (1 gene); Chloramphenicol O-acetyltransferases (4 genes) | 10 | chloramphenicol, (carbenicillin, erythromycin, novobiocin, streptomycin and tetracycline additionally for Antibiotic efflux pump transporters ArpB and ArpC) | 01590, 01447, 02000, 00372, 02994, 02096; 01076, 02396, 04311, 05724 |
| L-arabinose transferases and deformylase ArnD (genes cluster (5+1 genes)); Lipid A phosphoethanolamine transferase (2 genes); Histidine kinase; Transcriptional regulators (6 genes); Ribosomal protein S5]-alanine N-acetyltransferase (2 genes) | 17 | polymyxin and cationic antimicrobial peptides (CAMP) | 01385, 01386; 01387, 01388, 01389, 05003; 01616, 03042; 01852; 02135, 02314,02498, 03845, 04273, 04958; 00986, 00987 |
| Macrolide efflux and transport proteins | 6 | macrolides, lincosamides, streptogramin B analogs | 01455, 02271, 02596, 02597, 04765, 05266 |
| puromycin resistance proteins pur8 (2 genes); Multidrug resistance protein MdtN (2 genes); | 4 | Puromycin, teicoplanin | 01566, 02097, 02956, 03761 |
| Ribosomal protein S5]-alanine N-acetyltransferases | 2 | polymyxin B, colistin, puromycin, chloramphenicol | 00986, 00987 |
| undecaprenyl-diphosphate phosphatase | 1 | bacitracin | 03056 |
| Transferases, deformylase ArnD, peptide transporters | 7 | antimicrobial peptides | 01385, 01386, 01387, 01388, 01389 (gene cluster);  04578, 05003 |
| Streptomycin 6-kinase; streptomycin 3-adenylyltransferases (4 genes); antibiotic efflux pumps ArpB and ArpC (3 genes); Quorum-quenching N-acyl-homoserine lactonase | 9 | carbenicillin, chloramphenicol, erythromycin, novobiocin, streptomycin and tetracycline | 03157;  00035, 02259, 03422, 03514;  00372, 02994, 02096;  00729 |
| beta-lactamases and transcriptional activators | 19 | Methicillin, carbapenem, cephalosporin and other beta-lactams | 00820, 00821, 01324, 02018, 03205, 03222, 03698, 03840, 03981, 04019, 04244, 04289, 04465, 04595, 05762 |
| spermidine export proteins MdtJ (small multidrug resistance family) | 2 | Polyamines, small multidrugs | 03130, 03491 |
| protein DrgA | 1 | herbicide Dinoseb and metronidazole | 05433 |
| Multidrug export proteins EmrA | 5 | carbonyl cyanide m-chlorophenyl-hydrazone (CCCP); carbonyl cyanide 4-trifluoromethoxyphenylhydrazone, (FCCP); 2,4- dinitrophenol (DNP) and nalidixic acid | 01626, 01789, 03787, 05353, 05354 |
| multi drug resistance protein PmpM | 1 | benzalkonium chloride, fluoroquinolones, ethidium bromide, acriflavine and tetraphenylphosphonium chloride | 03524 |
| sodium-coupled monocarboxylate transporter | 1 | monocarboxylate drugs (nicotinate, benzoate, salicylate and 5-aminosalicylate) and iodide | 03695 |
| Quaternary ammonium compound-resistance proteins SugE (small multidrug resistance family) | 2 | cetylpyridinium, cetyldimethylethyl ammonium and cetrimide cations | 01664, 04242 |
| Ethidium bromide resistance proteins (small multidrug resistance family) | 3 | ethidium bromide and quaternary ammonium compounds; acriflavine and tetraphenylphosphonium chloride (03524) | 03490, 03524, 05181 |
| organic hydroperoxide resistance regulators | 3 | terta-butyl hydroperoxide, catechol | 02083, 02084, 02108 |
| arsenical resistance proteins | 3 | Arsenate | 02709, 02710, 04702 |
| Acid resistance proteins (histidine kinases (2 genes), | 12 | Acid stress and acidic compounds (acetic acid (high concentration), L-methionine sulfoximine (phosphinotricin), antibiotic mupirocin (pseudomonic acid A), acidic environment in the stomach (03199); acid anhydrides, deoxycholate; sodium dodecyl sulfate (SDS), and nalidixic acid (02448) | 05423, 05694, 05053, 04731, 04523, 03890, 03844, 03199, 02596, 02448, 02315, 01387 |
| Na(+)/H(+) antiporters (2 genes); salt stress proteins (8 genes); osmoregulators and protectors (40 genes) | 45 | Na(+) resistance, salt stress; osmotic stress | 03968, 03971; 01458, 01618, 05402; 01676, 04942, 04944, 05325, 02784 |
| Copper resistance proteins (copper resistance operon CopABCD) | 5 | Copper, silver (01262) | 01169, 01262, 02946, 02947, 03676 |
| Glutathione transferase | 1 | fosfomycin | 03896 |
| Cation efflux system operon (proteins CzcA, CzcB, CzcC, oxidoreductase. Histidine kinase) | 5 | Cobalt, zinc, cadmium | 02134, 02455, 02456, 02457, 05639 |
| Heat shock proteins (chaperons) | 13 | High temperature stress | 00321, 01643, 01800, 02103, 03433, 03890, 03959; 04942, 04943, 04944 (gene cluster), 05061, 05438 |
| Cold-shock proteins (activators, histidine kinase) | 6 | cold stress | 00400, 01458, 01663, 02349, 03865, 04060 |
| Organic hydroperoxide resistance proteins, peroxiredoxins (7 genes), Sarcosine oxidases (6 genes), dehydrogenases, phosphatases, transferases, histidine kinase | 19 | Oxidative stress (peroxides, H2O2, etc.) | 00305, 00595, 00900, 01643, 01761, 01957, 02183, 02254, 02714, 02779, 02811, 03406, 03995, 04180, 04190, 04406, 05307, 05351, 05436 |
| Toluene tolerance proteins; aromatic hydrocarbon degradation proteins (laccases, oxidoreductases, monooxygenase, dehydrogenases, decarboxylases, etc.) | 69 | Toluene (6 genes), phenols (19 genes), benzoates (33 genes), detergents (3 genes) etc. | 00733, 02054, 02458, 02482, 02484, 04525 |
| Ultra violet repair (10 UVr-related genes) and DNA anti-damaging systems | 74 | Uvr-induced dsDNA breaks and lesions | 00403, 00695, 01787, 01928, 02109, 02387, 04353, 04675, 05541, 05692 |
| Antiviral defense factors (CRISPR (clustered regularly interspaced short palindromic repeat) -associated gene cluster) | 7 | bacteriophages | 02555, 02556, 02557, 02558, 02559, 02560, 04258 |
